# Efficacy and Safety of First-line Systemic Therapy for Metastatic Renal Cell Carcinoma: A Systematic Review and Network Meta-analysis

**DOI:** 10.1016/j.euros.2021.12.007

**Published:** 2022-01-22

**Authors:** Nicholas A. Bosma, Matthew T. Warkentin, Chun Loo Gan, Safiya Karim, Daniel Y.C. Heng, Darren R. Brenner, Richard M. Lee-Ying

**Affiliations:** a Department of Oncology, University of Calgary Tom Baker Cancer Centre, Calgary, AB, Canada; b Lunenfeld-Tanenbaum Research Institute, Sinai Health System, Toronto, ON, Canada; c Dalla Lana School of Public Health, University of Toronto, Toronto, ON, Canada; d Department of Community Health Sciences, University of Calgary Cumming School of Medicine, Calgary, AB, Canada

**Keywords:** Immunotherapy, Renal cell carcinoma, Tyrosine kinase inhibitors, Network meta-analysis

## Abstract

**Context:**

Considerable advances have been made in the first-line treatment of metastatic renal cell carcinoma (mRCC), with immunotherapy-based combinations including immunotherapy-tyrosine kinase inhibitors (IO-TKIs) and dual immunotherapy (IO-IO) favored. A lack of head-to-head clinical trials comparing these treatments means that there is uncertainty regarding their use in clinical practice.

**Objective:**

To compare and rank the efficacy and safety of first-line systemic treatments for mRCC with a focus on IO-based combinations.

**Evidence acquisition:**

MEDLINE (Ovid), EMBASE, Cochrane Library, Web of Science, and abstracts of recent major scientific meetings were searched to identify the most up-to-date phase 3 randomized controlled trials (RCTs) of first-line IO-based combinations for mRCC up to June 2021. A systematic review and network meta-analysis were completed using the Bayesian framework. Primary endpoints included overall survival (OS) and progression-free survival (PFS). Secondary endpoints included the objective response rate (ORR), complete response (CR), grade 3–4 treatment-related adverse events (TRAEs), treatment-related drug discontinuation (TRDD), and health-related quality of life (HRQoL). The analysis was performed for the intention-to-treat (ITT) population as well as by clinical risk group.

**Evidence synthesis:**

A total of six phase 3 RCTs were included involving a total of 5121 patients. Nivolumab plus cabozantinib (NIVO-CABO) had the highest likelihood of an OS benefit in the ITT population (surface under the cumulative ranking curve 82%). Avelumab plus axitinib (AVEL-AXI) had the highest likelihood of an OS benefit for patients with favorable risk (65%). Pembrolizumab plus AXI (PEMBRO-AXI) had the highest likelihood of an OS benefit for patients with intermediate risk (78%). PEMBRO plus lenvatinib (PEMBRO-LENV) had the highest likelihood of an OS benefit for patients with poor risk (89%). PEMBRO-LENV was associated with a superior PFS benefit across all risk groups (89–98%). Maximal ORR was achieved with PEMBRO-LENV (97%). The highest likelihood for CR was attained with NIVO plus ipilimumab (NIVO-IPI; 85%) and PEMBRO-LENV (83%). The highest grade 3–4 TRAE rate occurred with PEMBRO-LENV (95%) and NIVO-CABO (83%), but the latter was associated with the lowest TRDD rate (2%). By contrast, NIVO-IPI had the lowest grade 3–4 TRAE rate (6%) and the highest likelihood of TRDD (100%). HRQoL consistently favored NIVO-CABO (66–75%), PEMBRO-LENV (44–85%), and NIVO-IPI (65–93%) in comparison to the other treatments.

**Conclusions:**

IO-TKI drug combinations are associated with consistent improvements in clinically relevant outcomes for all mRCC risk groups. This benefit may be at the cost of higher TRAE rates; however, lower TRDD rates suggest a manageable side-effect profile. Longer follow-up is required to determine if the benefits of IO-TKIs will be sustained and if they should be favored in the first-line treatment of mRCC.

**Patient summary:**

Combination treatments based on immunotherapy agents continue to show meaningful benefits in the first-line treatment of metastatic kidney cancer. Our review and network meta-analysis shows that immunotherapy combined with another class of agents called tyrosine kinase inhibitors is promising. However, longer follow-up is needed for this treatment strategy to clarify if the benefits are long-lasting.

## Take Home Message

Immunotherapy-tyrosine kinase inhibitor (IO-TKI) drug combinations are associated with consistent improvements in clinically relevant outcomes for all risk groups in metastatic renal cell carcinoma (mRCC). Longer follow-up is required to determine if the benefits of IO-TKI regimens will be sustained and if they should be favored in the first-line treatment of mRCC.

## 1 Introduction

Globally, new cases of renal cell carcinoma (RCC) in 2020 were diagnosed in approximately 430 000 patients, associated with 180 000 deaths [1]. Surgery is the mainstay of treatment for clinically localized disease; however, recurrence is common and is estimated to occur in 20–40% of patients [2], [3]. Furthermore, approximately 30% of new RCC cases present with advanced or metastatic disease (mRCC) for which curative-intent management is not possible [4].

Systemic therapy is the primary treatment strategy for mRCC and has evolved rapidly over the past several decades as we advance our understanding of the disease biology and pathogenesis. Clear cell RCC is the most common histological type, accounting for 75–85% of all RCC cases [5], and is characterized by alterations to the Von Hippel Lindau (*VHL*) gene, resulting in activation of angiogenic factors such as vascular endothelial growth factor (VEGF) [6]. This led to successful application of VEGF tyrosine kinase inhibitors (VEGF-TKIs) such as sunitinib [7] and pazopanib [8], [9] in treatment for mRCC. Furthermore, an appreciation of RCC as an immunogenic tumor emerged from the antitumor activity of cytokines such as interleukin 2 (IL-2) [10] and interferon alfa (IFNα) [11], [12], as well as the observation of spontaneous resolution of metastatic disease after removal of the primary RCC [13]. More recently, there has been a paradigm shift to the use of immunotherapy (IO)-based combinations, such as IO-TKI and IO-IO regimens, as a more effective targeted treatment strategy for mRCC [14], [15], [16], [17], [18].

Coinciding with these advances, prognostic risk-factor models for patients with advanced or metastatic RCC have been developed to help risk-stratify patients and support clinical guidelines in directing therapy. The Memorial Sloan Kettering Cancer Center (MSKCC) and International mRCC Database Consortium (IMDC) models use patient characteristics for risk stratification, such as time from diagnosis to treatment and Karnofsky performance status, as well as biochemical parameters including blood cell counts, calcium, and LDH [19], [20]. Historically, these models were developed in the era of frontline VEGF-TKI agents and their use in the age of IO-based combinations is less appreciated. In the past 4 yr alone, six new IO drug combinations have demonstrated significant clinical efficacy compared to the standard of care (SOC) of VEGF-TKI sunitinib monotherapy [14], [15], [16], [17], [18], [21]. Without head-to-head clinical trials of these different treatment options, we are reliant on indirect comparisons. Here we report an updated network meta-analysis (NMA) on the efficacy, safety, health-related quality of life, and patient-reported outcomes for first-line treatment of mRCC, with a focus on IO drug combinations and subgroup analysis by clinical risk group. A “living NMA” is in development with the goal of continuously and promptly updating the analysis as new data and longer follow-up become available.

## 2 Evidence acquisition

### 2.1 Search strategy and information sources

In line with the Preferred Reporting Items for Systematic Reviews and Meta-Analyses (PRISMA; Supplementary Table 1), a comprehensive search strategy was created and piloted in conjunction with dedicated Health Sciences and Knowledge Resource librarians. The MEDLINE (Ovid), EMBASE, Cochrane Library, and Web of Science databases were searched for patients receiving first-line IO-based combinations for advanced or metastatic RCC up to March 25, 2021. In addition, abstracts of recent major scientific meetings were screened to identify the most up-to-date relevant clinical trials up to June 2021. Our main search terms included: (first line therap* OR first line treat*) AND (immune checkpoint inhibitor OR immunotherapy) AND (renal cell carcinoma OR advanced renal cell carcinoma OR metastatic renal cell carcinoma). We restricted our search strategy to randomized phase 3 clinical trials and to the period from 2000 to date to avoid capturing clinically irrelevant treatment strategies. Supplementary Table 2 includes the search strategy and the results from Medline (OVID) as an example.

### 2.2 Eligibility criteria

The trials included for analysis fulfilled the following primary criteria: (1) the study population were adults with advanced or metastatic RCC, (2) randomly assigned to receive an IO-based systemic treatment in the first line, (3) compared to another first-line systemic treatment option, and (4) reported on clinically relevant outcomes, including overall survival (OS), progression-free survival (PFS), objective response rate (ORR), complete response (CR), treatment-related adverse events (TRAEs), treatment-related drug discontinuation (TRDD), and health-related quality of life (HRQoL). Previous systemic therapy for advanced or metastatic RCC was not permitted. We restricted studies to phase 3 RCTs and excluded observational studies, case reports, letters, editorials, and reviews.

### 2.3 Study selection

Records obtained from the information sources described above were screened in duplicate (N.B. and C.L.G.). The results from the search strategies were input into the online Covidence systematic review software and duplicates were removed. The initial title and abstract screen identified relevant publications for further review. Full-text screening and review were also completed in duplicate (N.B. and C.L.G.) according to the inclusion and exclusion criteria. Initial inter-rater conflict was resolved through consensus agreement and, when needed, expert consultation (R.L.Y., S.K., and D.H.).

### 2.4 Data extraction

A data extraction form was generated and piloted to collate information gathered from individual studies in a standardized way. Data extraction was completed independently and in duplicate (N.B. and C.L.G.) to ensure accuracy and agreement. Discrepancies were resolved via consensus agreement. If required, consensus was achieved via a co-author (R.L.Y., S.K., and D.H.). Data were extracted for the purpose of both a systematic review and NMA and included, but were not limited to: article information, eligibility checklist, study details, patient demographics, treatment characteristics, and outcome measures (Supplementary Table 3). Data were extracted from the most up-to-date trial analysis with the longest follow-up.

### 2.5 Quality assessment

The studies included underwent a quality assessment in duplicate (N.B. and C.L.G.) using the Cochrane Collaboration Tool for Assessment of Bias in Randomized Trials (Supplementary Table 4). Using this tool, studies were evaluated on the basis of their methods and description of random sequence generation, allocation concealment, selective reporting, blinding of participants and personnel, blinding of the outcome assessment, and risk of incomplete outcome data that could contribute to selection, reporting, performance, detection, attrition, or other sources of bias.

### 2.6 Statistical analysis

We fitted fixed-effect Bayesian NMAs to estimate the relative treatment effects for all possible pairwise treatments according to the studies included as described above. NMAs combine both direct and indirect evidence to estimate treatment effects. We performed meta-analysis of multiple study-reported outcomes reported as hazard ratios (HRs), including OS, PFS, and HRQoL metrics (EuroQol 5-Dimension [EQ-5D] and Functional Assessment of Cancer Therapy-Kidney Symptom Index [FKSI] questionnaires), and of outcomes reported as proportions, including ORR, CR, TRAEs of any grade or grade 3–4, and TRDD. Outcomes were analyzed on and intention to treat (ITT) basis and by prognostic subgroups (favorable, intermediate, and poor risk).

For outcomes reported as HRs, meta-analysis was performed on the log-HR scale using a Gaussian likelihood and identity link function to estimate the HR and 95% credible interval (CrI). For outcomes reported as proportions, meta-analysis was performed using a binomial likelihood and logit link function and reported as the odds ratio (OR) and 95% CrI. NMAs were fitted using the Markov chain Monte Carlo method with Gibbs sampling. Each NMA was fitted using four chains, each with 20 000 samples and 5000 samples as burn-in. For each outcome, we report treatment ranks (probabilities) and the surface under the cumulative ranking curve (SUCRA) to assess treatment optimality. Point estimates and CrIs for all pairwise treatments are reported. All statistical analyses were performed using R v4.0.5 (R Foundation for Statistical Computing, Vienna, Austria) [22] and the *gemtc* package [23].

## 3 Evidence synthesis

### 3.1 Search results and study selection

**Fig. 1:**
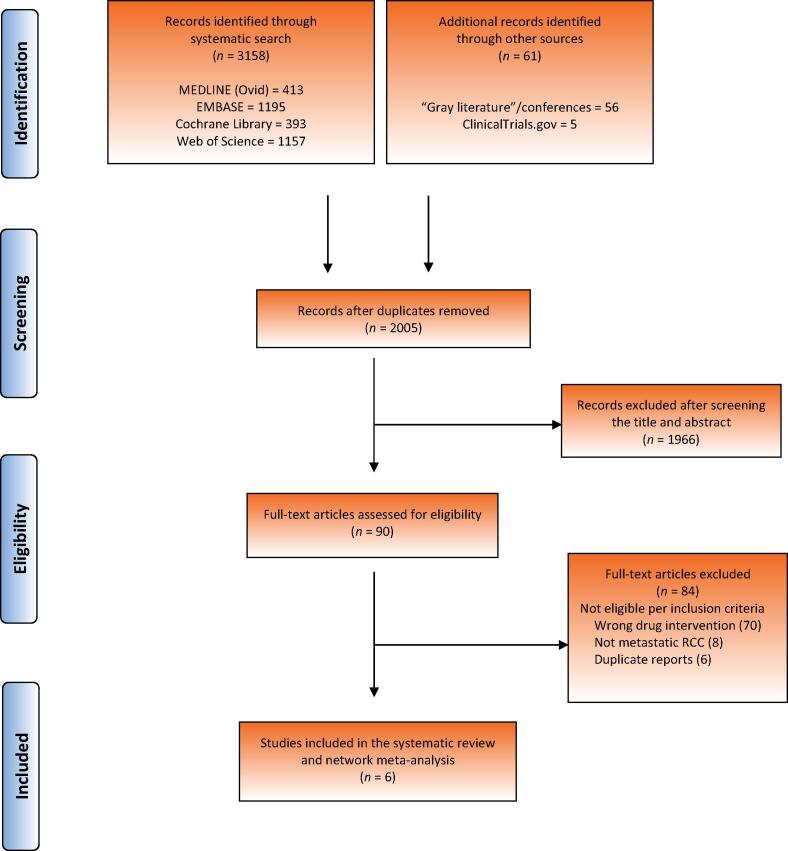
Preferred Reporting Items for Systematic Reviews and Meta-Analyses (PRISMA) flow diagram for the literature search and study selection. RCC = renal cell carcinoma.

The literature search yielded 3158 potential eligible studies, with an additional 61 records identified through other sources, such as recent oncological conferences. After removal of duplicates, 2005 articles underwent abstract and title screening, of which 90 were identified for full-text review. The main reason for exclusion was not meeting eligibility criteria, in particular studies that included the wrong drug intervention without an IO-drug combination. A total of six phase 3 RCT studies were included in the systematic review and NMA. The search results and study selection process is summarized in the PRISMA flow diagram in Fig. 1.

### 3.2 Study characteristics

The main characteristics of the six RCTs included are summarized in Table 1. A total of 5121 patients were included in the NMA. All of the trials had a similar two-arm randomized study design with sunitinib as the common comparator. The demographic characteristics of patients were well balanced, with a predominantly male population (70–78%) with a median age of 61–64 yr. Median follow-up ranged from 18.1 to 55 mo. CheckMate 214 (nivolumab [NIVO]-ipilimumab [IPI]) has the longest follow-up (55 mo) and CheckMate 9ER (NIVO-cabozantinib [CABO]) is the most recent trial with the shortest median follow-up (18.1 mo). According to MSKCC or IMDC risk criteria, there were a total of 1267 (24.7%), 3105 (60.6%), and 737 (14.4%) patients in the favorable, intermediate, and poor risk groups, respectively. A higher percentage of favorable risk patients were observed in Keynote 426 (pembrolizumab [PEMBRO]-axitinib [AXI]) and CLEAR (PEMBRO-lenvatinib [LENV]) at 31–35% when compared to the other RCTs (20–23%). By contrast, a higher percentage of poor risk patients were identified in CheckMate 214, Javelin 101 (avelumab [AVEL]-AXI), and CheckMate9ER (16–21%) when compared to the other trials (9–13%). IMmotion 151 (atezolizumab [ATEZO]-bevacizumab [BEV]) had more intermediate risk patients (69%) relative to the other RCTs (54–62%).

**Table 1:** Characteristics and summary of results for the studies of first-line treatment of metastatic renal cell carcinoma included in the network meta-analysis

Trial	ITT (*n*)	Patients by risk group, *n* (%)			Male (%)	Median Age Years (IQR)	Median Follow-Up (months)	Median OS (months)	Median PFS (months)	Response (%)	
		Favorable	Intermediate	Poor						ORR	CR
CheckMate 214 [14], [35], [36]											
Nivolumab + Ipi	550	125 (23)	334 (61)	91 (17)	75	62 (26-85)	55	NE	12.2	39.1	10.7
Sunitinib	546	124 (23)	333 (61)	89 (16)	72	62 (21-85)		38.4	12.3	32.4	2.6
								**HR 0.69 (0.59–0.81)**	HR 0.89 (0.76–1.05)		
Javelin 101 [15], [47]											
Avelumab + axitinib	442	94 (21)	271 (61)	72 (16)	72	62 (29-83)	19	NE	13.3	52.5	3.8
Sunitinib	444	96 (22)	276 (62)	71 (16)	78	61 (27-88)		NE	8.0	27.3	2.0
								HR 0.80 (0.62–1.03)	**HR 0.69 (0.57–0.83)**		
CheckMate 9ER [18]											
Nivolumab + Cabo	323	74 (23)	188 (58)	61 (19)	77	62 (29-90)	18.1	NE	16.6	55.7	8.0
Sunitinib	328	72 (22)	188 (58)	68 (21)	71	61 (28-86)		NE	8.3	27.1	4.6
								**HR 0.60 (0.40–0.89)**	**HR 0.51 (0.41–0.64)**		
Keynote 426 [16], [32]											
Pembro + axitinib	432	138 (32)	238 (55)	56 (13)	71	62 (30-89)	30.6	NE	15.4	60	9
Sunitinib	429	131 (31)	246 (57)	52 (12)	75	61 (26-90)		35.7	11.1	40	3
								**HR 0.68 (0.55–0.85)**	**HR 0.71 (0.60–0.84)**		
IMmotion 151 [21]											
Atezo + Bev	454	89 (20)	311 (69)	54 (12)	70	62 (56-69)	24	33.6	11.2	37	5
Sunitinib	461	90 (20)	318 (69)	53 (12)	76	60 (54-66)		34.9	8.4	33	2
								HR 0.93 (0.76–1.14)	**HR 0.83 (0.70–0.97)**		
CLEAR [17]											
Pembro + lenvatinib	355	110 (31)	210 (59)	33 (9)	72	64 (34-88)	26.6	NE	23.9	71.0	16.1
Sunitinib	357	124 (35)	192 (54)	37 (10)	77	61 (29-82)		NE	9.2	36.1	4.2
								**HR 0.66 (0.49–0.88)**	**HR 0.39 (0.32–0.49)**		

ITT = intention-to-treat population; OS = overall survival; PFS = progression free survival; ORR = objective response rate; CR = complete response; IQR = interquartile range; NE = not estimable; HR = hazard ratio (95% confidence interval), with significant results in bold; Ipi = ipilimumab; Cabo = cabozantinib; Pembro = pembrolizumab; Atezo = atezolizumab; Bev = bevacizumab.

### 3.3 Network meta-analysis

The networks of eligible comparisons for the outcomes of interest are graphically represented in Supplementary Fig. 1. A total of seven different systemic treatments were included in the NMA, six of which were IO-drug combinations versus the common comparator of sunitinib monotherapy. Graphical summaries of the OS and PFS HR results for each of the clinical trials for the ITT and risk group populations are shown in Supplementary Fig. 2.

#### 3.3.1 Overall survival

**Fig. 2:**
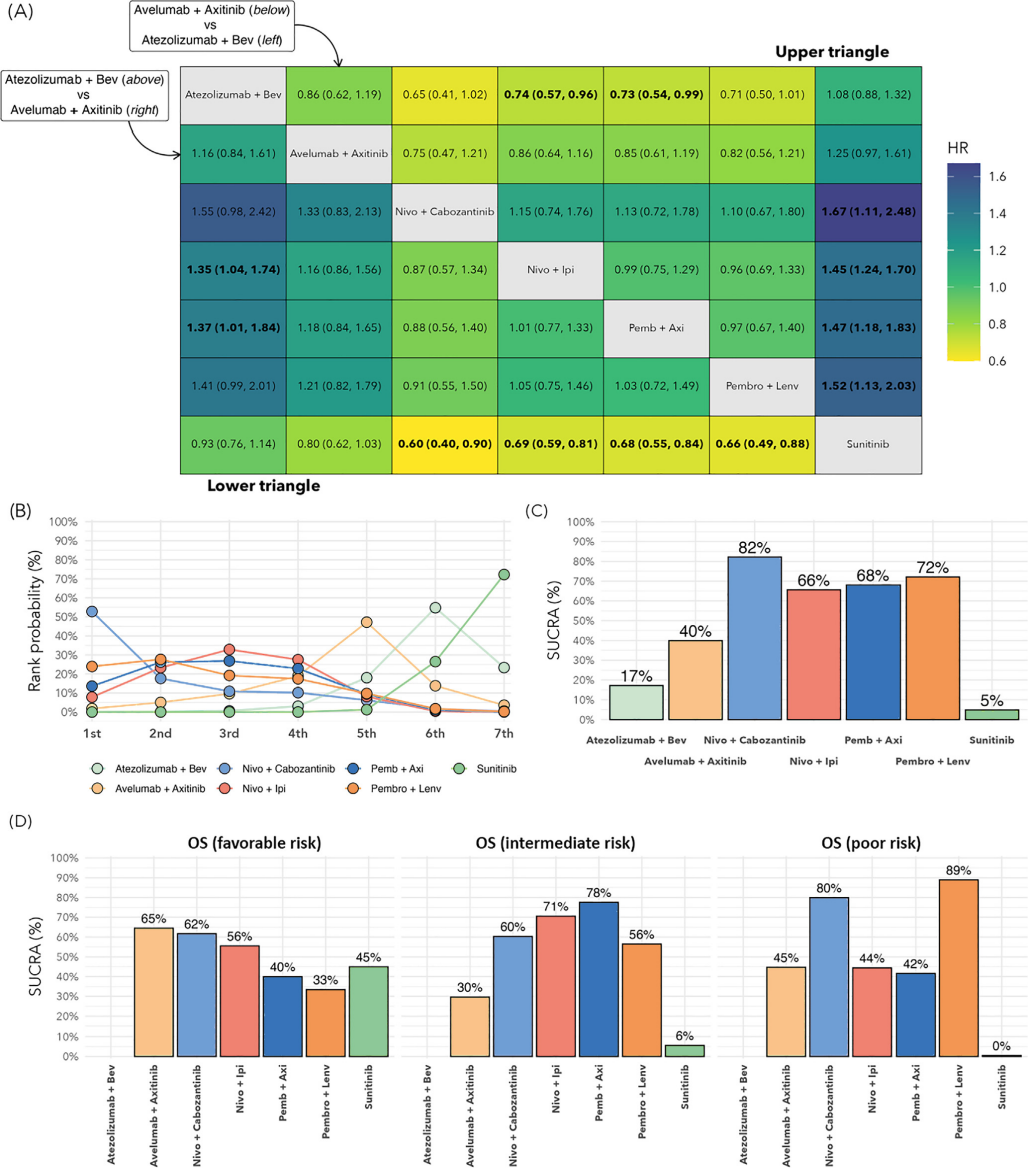
OS results for the ITT population and by clinical risk group. (A) HRs for OS for the ITT population. Treatment strategies separate the table diagonally to present pairwise comparisons of all systemic therapies. HRs in the upper triangle portion of the table are for comparison of the treatment below against the treatment to the left. In the lower triangle, comparisons are made between the treatment above and the treatment to the right. Values in bold font are statistically significant. (B) Rankogram for OS in the ITT population. The *x*-axis indicates the probability of the preferential treatment being ranked in *n*th position. (C) SUCRA plot for OS for the ITT population, representing the overall ranking probability for each treatment. (D) SUCRA plots for OS by clinical risk group. ITT = intention to treat; OS = overall survival; HR = hazard ratio; SUCRA = surface under the cumulative ranking curve; Bev = bevacizumab; Nivo = nivolumab; Ipi = ipilimumab; Axi = axitinib; Pemb/Pembro = pembrolizumab; Lenv = lenvatinib.

OS data were available for all six trials included for the ITT population. Compared to sunitinib, better OS was observed with NIVO-CABO (HR 0.60, 95% CrI 0.40–0.90), NIVO-IPI (HR 0.69, 95% CrI 0.59–0.81), PEMBRO-AXI (HR 0.68, 95% CrI 0.55–0.84), and PEMBRO-LENV (HR 0.66, 95% CrI 0.49–0.88; Fig. 2A). Analysis of treatment ranking revealed that NIVO-CABO (SUCRA 82%) had the highest likelihood of being the preferred treatment option compared to the other treatment strategies in the ITT population (Fig. 2B,C).

The OS analysis by risk group is shown in Fig. 2D. OS data for ATEZO-BEV were not available by risk group. In the favorable risk group, AVEL-AXI had the highest likelihood of being the preferred treatment option for OS (SUCRA 65%), followed by NIVO-CABO (SUCRA 62%) and NIVO-IPI (SUCRA 56%). For the intermediate risk group, PEMBRO-AXI had the highest likelihood of being the preferred treatment option (SUCRA 78%), followed by NIVO-IPI (SUCRA 71%) and NIVO-CABO (SUCRA 60%). For the poor risk group, both PEMBRO-LENV (SUCRA 89%) and NIVO-IPI (SUCRA 80%) were associated with the highest likelihood of being the preferred treatment.

#### 3.3.2 Progression-free survival

**Fig. 3:**
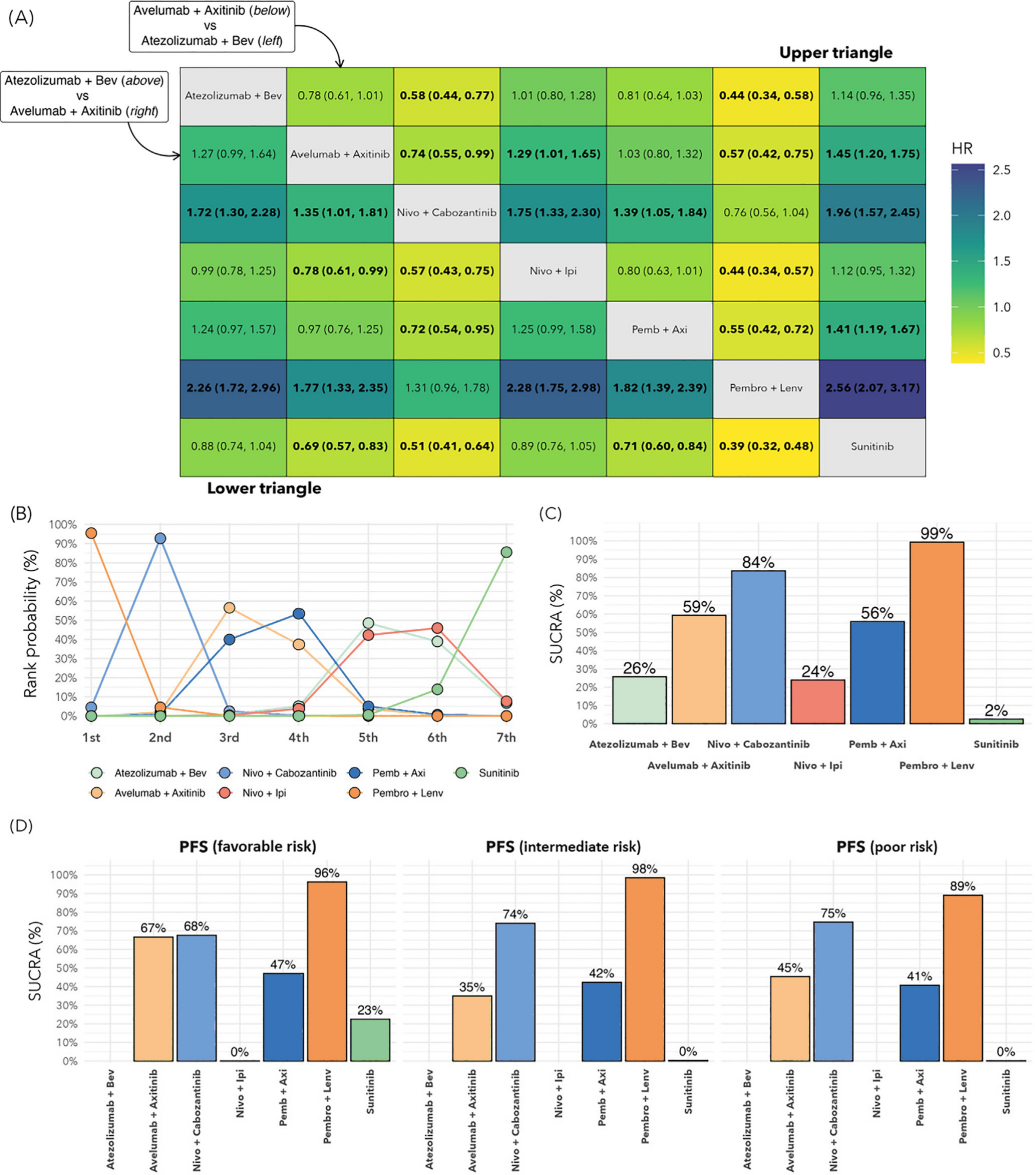
PFS results for the ITT population and by clinical risk group. (A) HRs for PFS for the ITT population. Treatment strategies separate the table diagonally to present pairwise comparisons of all systemic therapies. HRs in the upper triangle of the table are for comparison of the treatment below against the treatment to the left. In the lower triangle, comparisons are made between the treatment above and the treatment to the right. Values in bold font are statistically significant. (B) Rankogram for PFS in the ITT population. The *x*-axis indicates the probability of the preferential treatment being ranked in *n*th position. (C) SUCRA plot for PFS for the ITT population, representing the overall ranking probability for each treatment. (D) SUCRA plots for PFS by clinical risk group. ITT = intention to treat; PFS = progression free survival; HR = hazard ratio; SUCRA = surface under the cumulative ranking curve; Bev = bevacizumab; Nivo = nivolumab; Ipi = ipilimumab; Axi = axitinib; Pemb/Pembro = pembrolizumab; Lenv = lenvatinib.

In the ITT population, PFS data were available from all six trials included. Compared to sunitinib, better PFS was observed with AVEL-AXI (HR 0.69, 95% CrI 0.57–0.83), NIVO-CABO (HR 0.51, 95% CrI 0.41–0.64), PEMBRO-AXI (HR 0.71, 95% CrI 0.60–0.84), and PEMBRO-LENV (HR 0.39, 95% CrI 0.32–0.48; Fig. 3A). Analysis of treatment ranking revealed that PEMBRO-LENV (SUCRA 99%) followed by NIVO-CABO (SUCRA 84%) had the highest likelihood of being the preferred treatment compared to the other treatment strategies in the ITT population (Fig. 3B,C).

The PFS analysis by risk group is shown in Fig. 3D. PFS data for ATEZO-BEV and NIVO-IPI were not available. PEMBRO-LENV was consistently associated with the highest likelihood of being the preferred treatment option regarding PFS across favorable, intermediate, and poor risk subgroups (SUCRA 96%, 98%, and 89%, respectively).

#### 3.3.3 Objective response rate

**Fig. 4:**
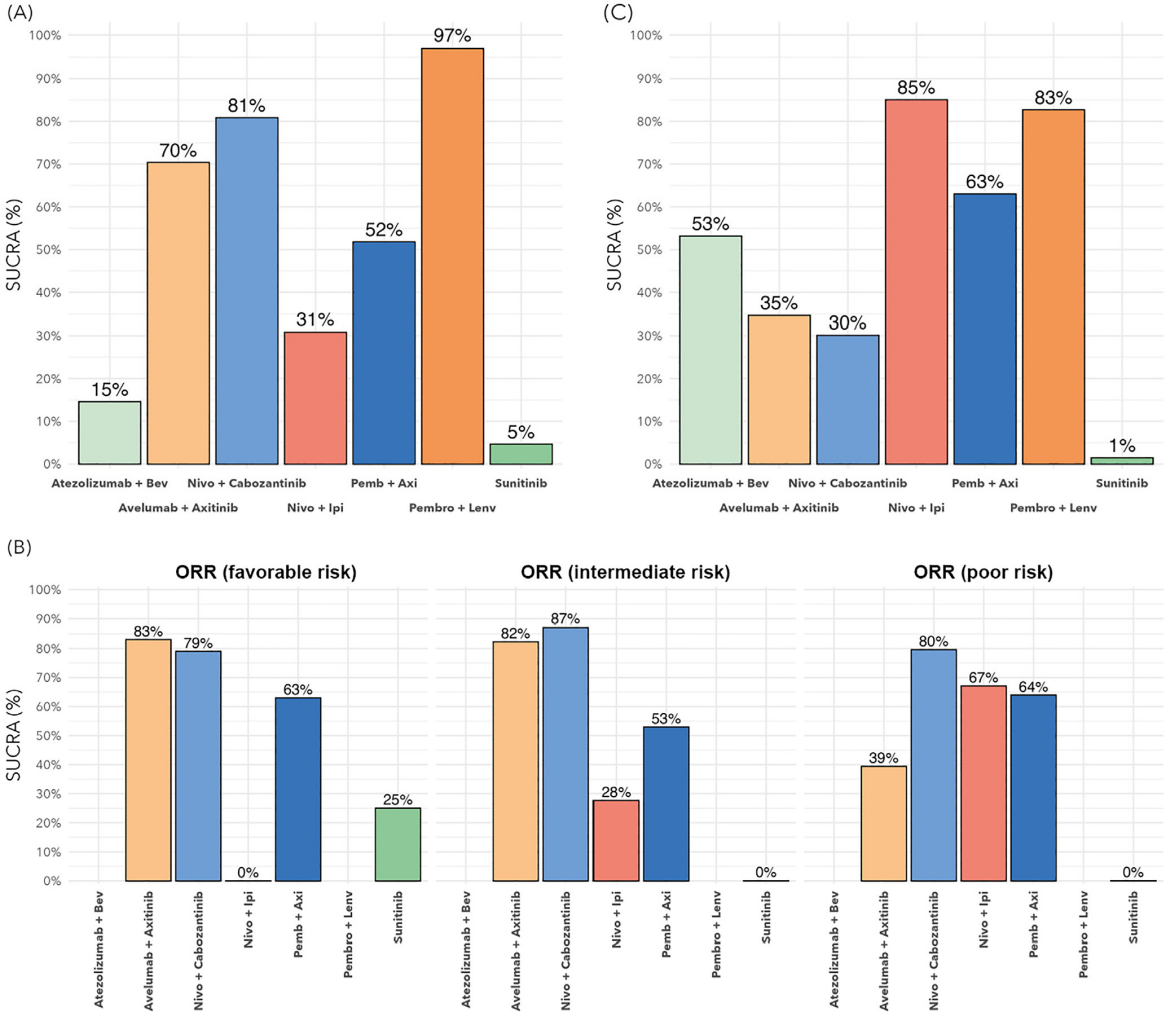
SUCRA plots of response rates for the ITT population and by clinical risk group, representing the overall preferential ranking probability for each treatment. (A) ORR for the ITT population. (B) ORR by clinical risk group. (C) CR by ITT. ITT = intention to treat; ORR = objective response rate; CR = complete response; HR = hazard ratio; SUCRA = surface under the cumulative ranking curve; Bev = bevacizumab; Nivo = nivolumab; Ipi = ipilimumab; Axi = axitinib; Pemb/Pembro = pembrolizumab; Lenv = lenvatinib.

For ORR analysis, all six trials were included in the NMA for the ITT population. Compared to sunitinib, all of the IO-drug combinations demonstrated higher odds for better ORR, with the exception of ATEZO-BEV (Supplementary Fig. 5). The highest odds for ORR were observed for PEMBRO-LENV (OR 4.36, 95% CrI 3.19–6.00) and NIVO-CABO (OR 3.39, 95% CrI 2.45–4.72). Analysis of treatment ranking in the network revealed that PEMBRO-LENV (SUCRA 97%) was associated with the highest likelihood of being the preferred treatment regarding ORR, followed by NIVO-CABO (SUCRA 81%; Fig. 4A).

There were no ORR data by risk group available for ATEZO-BEV or PEMBRO-LENV. For the favorable risk group, AVEL-AXI (SUCRA 83%) had the highest likelihood of being the preferred treatment option, followed by NIVO-CABO (SUCRA 79%). In the intermediate and poor risk groups, NIVO-CABO had the highest likelihood of being the preferred treatment option (SUCRA 87% and 80%, respectively; Fig. 4B).

#### 3.3.4 Complete response

All six trials were included in the NMA for CR in the ITT population. NIVO-IPI (SUCRA 85%) and PEMBRO-LENV (SUCRA 83%) were associated with the highest likelihood of being the preferred treatment option regarding CR (Fig. 4C).

#### 3.3.5 Adverse events and drug discontinuation

**Fig. 5:**
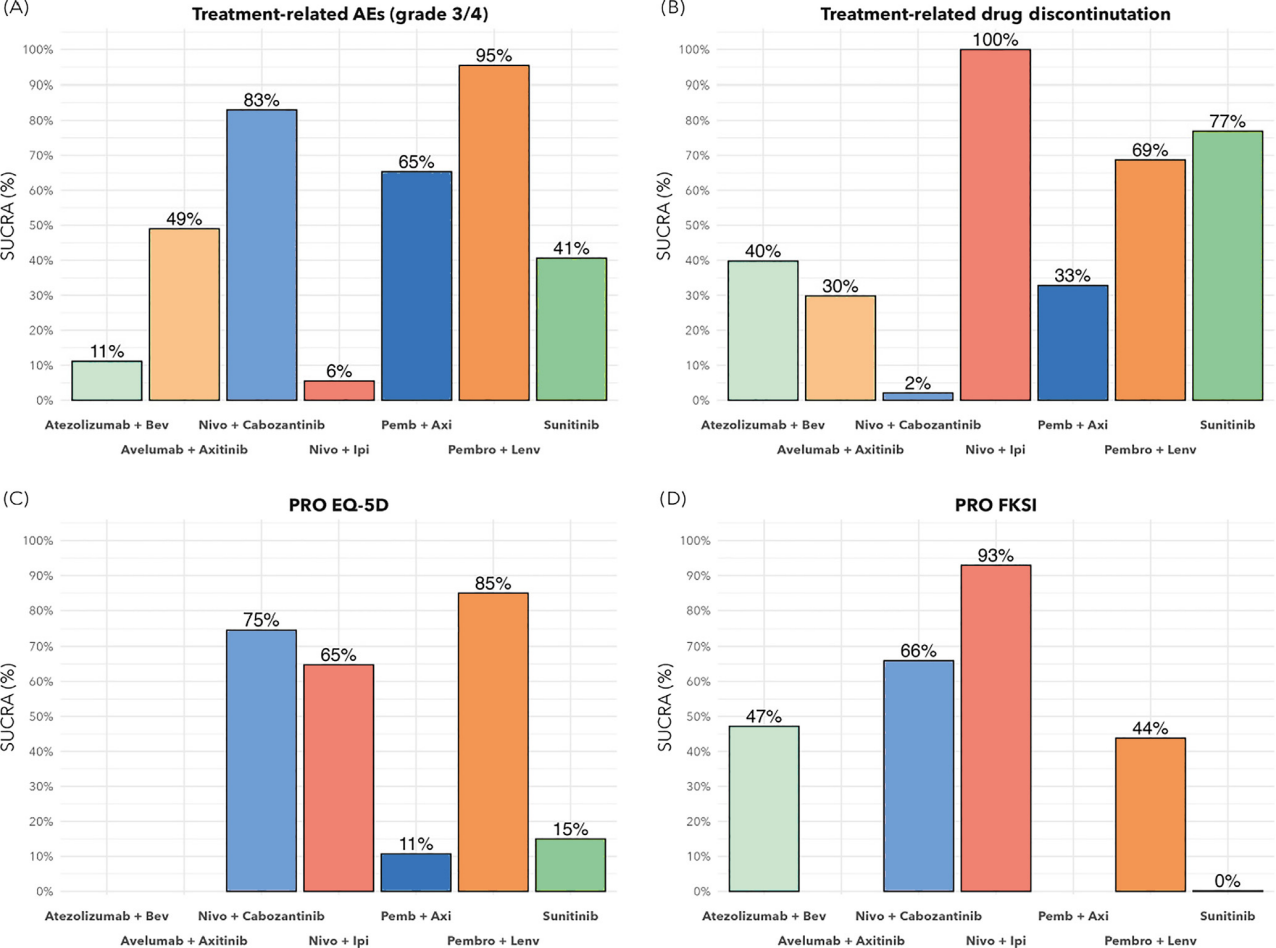
SUCRA plots of safety and health-related quality of life for the intention-to-treat population. (A) Treatment-related grade 3–4 adverse events and (B) treatment-related drug discontinuation, representing the overall ranking probability of an occurrence with the treatment strategy. Health-related quality of life, as measured with patient-reported outcome (PRO) questionnaires (C) EuroQol 5-Dimension (EQ-5D) (C) and (D) Functional Assessment of Cancer Therapy-Kidney Symptom Index (FKSI), representing the preferential ranking probability for each treatment. SUCRA = surface under the cumulative ranking curve; Bev = bevacizumab; Nivo = nivolumab; Ipi = ipilimumab; Axi = axitinib; Pemb/Pembro = pembrolizumab; Lenv = lenvatinib.

Data were available from all six trials in the network for grade 3–4 TRAEs and TRDD. Compared to sunitinib, PEMBRO-LENV (OR 1.77, 95% CrI 1.29–2.43) and NIVO-CABO (OR 1.48, 95% CrI 1.08–2.04) were associated with significant grade 3–4 TRAEs, whereas NIVO-IPI (OR 0.52, 95% CrI 0.41–0.67) and ATEZO-BEV (OR 0.56, 95% CrI 0.43–0.73) were associated with the lowest odds for this outcome (Supplementary Fig. 6). Treatment ranking analysis revealed that PEMBRO-LENV (SUCRA 95%) followed by NIVO-CABO (SUCRA 83%) had the highest likelihood of being associated with grade 3–4 TRAEs when compared to the other agents (Fig. 5A).

Despite a lower rate of grade 3–4 TRAEs, NIVO-IPI was associated with the highest odds of TRDD compared to sunitinib (OR 1.99, 95% CrI 1.45–2.76; Supplementary Fig. 7). This finding is consistent with the treatment ranking analysis: NIVO-IPI was associated with the highest likelihood of TRDD (SUCRA 100%) when compared to the other agents. By contrast, despite a higher rate of grade 3–4 TRAEs, NIVO-CABO was associated with the highest likelihood of having the lowest TRDD rate (SUCRA 2%; Fig. 5B).

#### 3.3.6 HRQoL and patient-reported outcomes

Patient-reported outcomes (PROs) were available for the EQ-5D and FKSI questionnaires as measures of HRQoL. EQ-5D data were not available for ATEZO-BEV and AVEL-AXI, and FKSI data were not available for AVEL-AXI and PEMBRO-AXI. Analysis of treatment ranking revealed that PEMBRO-LENV (SUCRA 85%) followed by NIVO-CABO (SUCRA 75%) were associated with the highest likelihood of being the preferred treatment regarding EQ-5D (Fig. 5C). For the FKSI questionnaire, NIVO-IPI (SUCRA 93%) followed by NIVO-CABO (SUCRA 66%) was associated with the highest likelihood of being the preferred treatment (Fig. 5D).

#### 3.3.7 Publication bias

There was slight asymmetry of the funnel plot, with the most recent trials (CheckMate 9ER [NIVO-CABO] and CLEAR [PEMBRO-LENV]) demonstrating larger standard error for the log-HR; however, limited by the number of studies, there was no clearly detectable publication bias (Supplementary Fig. 8).

### 3.4 Discussion

The treatment landscape for mRCC is rapidly changing, with IO drug combinations now being favored. There is a lack of head-to-head trials evaluating these different treatment strategies. We performed a systematic review and NMA to provide a quantitative indirect treatment comparison with a focus on IO combinations and clinical risk subgroups. Our analysis explored clinically relevant efficacy outcomes including OS, PFS, ORR, and CR, and grade 3–4 TRAEs, TRDD, and HRQoL.

The NMA demonstrated a trend for better clinical outcomes with IO-TKI combinations. Treatment ranking analysis for the primary endpoints of OS and PFS in the ITT population identified the IO-TKI combinations of NIVO-CABO and PEMBRO-LENV as the most preferred agents. Emerging evidence indicates a strong interplay between the immune system and angiogenesis [24], which provides strong biological validation for the use of IO-TKI combinations. For example, proangiogenic factors have been identified in the suppression of T-cell differentiation, priming, and trafficking into the tumor microenvironment, as well as upregulation of PD-L1 on tumor cells [25]. In this regard, IO-TKI combinations may more effectively target the immune system, endothelium, and tumor cells simultaneously [26]. However, there is increasing evidence to suggest the existence of biological or molecular clusters within RCC that display a more predominant angiogenic or immunogenic phenotype that may predict sensitivity to inhibitors of angiogenesis and/or immunotherapy [27]. In the phase 2 IMmotion 150 trial, higher efficacy with the VEGFR inhibitor sunitinib was observed in tumors with high angiogenesis gene expression, whereas high immune or low angiogenesis gene expression were associated with better clinical benefit with ATEZO-BEV. Another interesting finding was the association of high angiogenesis gene expression and favorable MSKCC risk group compared to intermediate to poor risk groups [28]. It has also been shown that utilization of gene signatures can improve the prognostic predictive power of the IMDC risk model [29]. The incorporation of molecular clusters in clinical risk groups holds the potential to improve the classification of advanced RCC and help in guiding treatment decisions.

For the time being, risk classification using clinical characteristics alone, such as the IMDC model, represents the best tool for obtaining prognostic and predictive information. In our NMA, similar to results for the ITT population, IO-TKI combinations were favored for OS and PFS across the three clinical risk groups. The most preferred IO-TKI combination for OS benefit varied according to risk group, whereas PEMBRO-LENV and NIVO-CABO were consistently ranked highest for PFS in all risk groups. Updated guidelines for the first-line management of clear cell mRCC from the National Comprehensive Cancer Network and European Society of Medical Oncologists now suggest combination IO-TKI or TKI monotherapy (sunitinib or pazopanib) for favorable risk patients and IO-TKI or dual IO-IO (NIVO-IPI) for intermediate/poor risk patients [30]. The inclusion of IO-TKI combinations for all risk groups is a result of the rapid dissemination of clinical trials demonstrating better outcomes in the overall ITT population when compared to the prior SOC of sunitinib. There is currently controversy regarding whether IO-TKI combinations should be favored over sunitinib monotherapy for favorable risk patients. Similarly, it is unclear if IO-TKI combinations should be considered in the first line over NIVO-IPI for the treatment of intermediate/poor risk patients.

Our analysis corroborates a possible association of better outcomes with IO-TKI combinations over TKI monotherapy and dual IO-IO combinations regardless of risk group; however, this finding should be interpreted with caution. There is significant variability in the design of trials of IO drug combinations. The majority of trials investigating IO drug combinations were powered according to the ITT population and not risk groups, with the exception of CheckMate 214, which investigated OS, PFS, and ORR as primary endpoints with the IO-IO agents NIVO-IPI in the intermediate/poor risk group [14]. Therefore, it is difficult to make any conclusive treatment decisions from risk subgroup analyses alone. In fact, there have been no IO drug combinations that have demonstrated an OS benefit in the favorable risk group, questioning whether sunitinib monotherapy should still be considered the mainstay of treatment [31]. Moreover, PEMBRO-AXI, which has the longest follow-up for any IO-TKI agents, has continued to demonstrate worsening of HRs for OS in the favorable risk group over time from 0.64 [16] to 1.06 [32] and most recently 1.17 (95% confidence interval [CI] 0.76–1.80) [33] from the updated analysis presented at the 2021 American Society of Clinical Oncology conference. Longer follow-up will allow assessment of whether this trend occurs across all of the IO-TKI drug combinations for favorable risk patients. For intermediate/poor risk patients, trials of IO-TKI and IO-IO agents continue to demonstrate clinically meaningful benefits; however, the durability of the former treatment approach remains to be clarified. Real-world data suggest no difference in OS, PFS, or response rate between IO-TKI and NIVO-IPI as first-line treatment in the ITT population or intermediate/poor risk group, although use of IO-TKI in the first line may impede the response rate to SOC VEGF-TKIs in the second line [34]. Our NMA demonstrated a preference for IO-TKI agents in the intermediate or poor risk groups, particularly with the newest combinations of NIVO-CABO and PEMBRO-LENV. Although these are promising early results favoring IO-TKI agents across all risk groups, these trials have the least follow-up and it will remain to be seen if these benefits persist over time.

The CheckMate 214 trial, investigating dual IO-IO (NIVO-IPI) therapy, has the longest median follow-up of 55 mo, in contrast to the recently released 42-mo follow-up for the IO-TKI combination PEMBRO-AXI in Keynote 426. NIVO-IPI has continued to demonstrate durable OS benefits in the ITT population, with a sustained 30% reduction in deaths (OS HR 0.69, 95% CrI 0.59–0.81) [14], [35], [36] throughout the follow-up period. By contrast, PEMBRO-AXI has shown a slightly worse trend in the HR for OS over this time period from a 47% reduction in death (OS HR 0.53, 95% CI 0.38–0.74) [16] to 32% (OS HR 0.68, 95% CI 0.55–0.85) [32] to 27% (OS HR 0.73, 95% CI 0.60–0.88) [33] in the most recent follow-up analysis. Whereas the survival curves appear to have plateaued for NIVO-IPI, the long-term durability of PEMBRO-AXI and other IO-TKIs is still in question in the ITT populations and across clinical risk groups.

Some trials were designed and powered with stratification by PD-L1 expression ≥1%, such as Javelin 101 (AVEL-AXI) and IMmotion 151 (ATEZO-BEV), which puts into question the generalizability of their results [15], [21]. Although PD-L1 status has been used as a predictive biomarker in other cancers, the utility of this approach has not been identified in mRCC [37]. A recent meta-analysis identified a subset of PD-L1-positive mRCC patients who would derive a PFS benefit from IO in comparison to PD-L1-negative patients, but this was not observed for OS [38]. Furthermore, the heterogeneity of PD-L1 testing and analysis with various antibodies against tumor versus immune cell populations makes the generalizability of this biomarker unknown [37]. We performed an additional subgroup analysis by PD-L1 status; however, without data available for NIVO-IPI in the CheckMate 214 trial, accurate interpretation is not possible. CheckMate 214 only included an analysis stratified by PD-L1 ≥1% versus <1% in the intermediate/poor risk group and not the overall population [14] and therefore we were unable to include the trial. In general, our analysis demonstrated better OS and PFS for IO-TKI combinations compared to sunitinib, regardless of PD-L1 status (Supplementary Fig. 9). However, this finding is difficult to interpret given the inability to include NIVO-IPI and the lack of evidence regarding PD-L1 as a reliable predictive biomarker in mRCC.

Secondary endpoints in the NMA also favored IO-TKI combinations. PEMBRO-LENV was identified as the most preferred treatment option regarding ORR in the ITT population, followed by NIVO-CABO. NIVO-CABO was a preferred treatment option across all risk subgroups, whereas the CLEAR trial (PEMBRO-LENV) did not have ORR data available by risk subgroup. A relatively higher proportion of favorable risk patients in the CLEAR trial (31–35% vs 21–23% in other trials) may help to explain the higher ORR, as IO-TKI combinations have consistently demonstrated significant activity in this clinical risk subgroup, with response rates up to 70% [15], [16], [17], [18]. Interestingly, maximal CR was identified with NIVO-IPI in the overall population, consistent with the proportion of patients who experience durable responses from dual IO-IO treatments.

Clinically meaningful benefits from treatment strategies also need to be balanced against the associated side effects and toxicity. We investigated grade 3–4 TRAEs and TRDD as the most relevant clinical indicators of treatment tolerance and toxicity. In general, IO-TKIs had higher rates of grade 3–4 TRAEs, in particular PEMBRO-LENV and NIVO-CABO, whereas NIVO-IPI had the lowest rate. Paradoxically, TRDD was highest with NIVO-IPI, whereas NIVO-CABO had the lowest TRDD. This suggests that the treatment-related toxicity secondary to IO-TKIs is manageable and does not absolutely necessitate discontinuation. Furthermore, it suggests that there is probably a smaller proportion of patients with clinically significant treatment-related toxicity with NIVO-IPI, but when this occurs it is significant enough to necessitate drug discontinuation, presumably from immune-related adverse events. The highest chances of immune-related adverse events are during the 12-wk period of dual IO-IO treatment before transitioning to single-agent maintenance with NIVO. In general, the clinical trials reported lower corticosteroid requirements with IO-TKI (approx. 16%) compared to NIVO-IPI (approx. 29%), defined as ≥40 mg daily prednisone equivalent for any period of time. This observation is consistent with higher significant immune-related adverse events with NIVO-IPI that probably require drug discontinuation.

PRO questionnaires are increasingly being used as reliable indicators of HRQoL and risk of deterioration from treatment [39]. Data for the EQ-5D and FKSI health questionnaires were available for the NMA. The most recent IO-TKIs (NIVO-CABO and PEMBRO-LENV) and NIVO-IPI were consistently ranked as preferred treatments regarding both the EQ-5D and FKSI PRO questionnaires; however, there were missing data for PEMBRO-AXI and ATEZO-BEV, and no PRO data for AVEL-AXI. According to EQ-5D data, PEMBRO-LENV and NIVO-CABO were associated with the lowest risk of treatment-related deterioration, whereas NIVO-IPI was favored according to FKSI data. Heterogenity in the health questionnaires used to determine HRQoL and the inconsistent reporting across trials posed a challenge in effectively comparing treatment strategies. Furthermore, there is variable follow-up time between the trials for HRQoL. Checkmate 214 (NIVO-IPI) reported HRQoL with the longest follow-up of 103 wk, whereas the shortest follow-up was 30 wk in the Keynote-426 trial (PEMBRO-AXI). However, within the constraints of the available data, the NMA suggests an association of better HRQoL with the newer IO-TKI drug combinations PEMBRO-LENV and NIVO-CABO as well as NIVO-IPI.

It is important to note that other recently published NMAs have identified similar findings of promising activity with IO-TKI combinations, in particular NIVO-CABO and PEMBRO-LENV [40], [41]. Our NMA represents the most up-to-date analysis and continues to demonstrate this trend. Furthermore, the primary goals of our study were to provide concise and easy-to-interpret graphical and tabular representations of treatment efficacy to aid in clinical decision-making, and to create a platform for the future development of an interactive database with a “living NMA”. In addition, other unique features of this NMA are the inclusion of HRQoL analysis and examination of results for clinical risk groups separately in terms of favorable, intermediate, and poor risk. This latter exploration was conducted to assess whether treatment strategies should be tailored to each independent clinical risk group rather than the current dichotomous classification of favorable versus intermediate/poor risk. There was an observation of preferential benefit with NIVO-CABO or PEMBRO-LENV in the poor-risk patient population, whereas a more variable distribution was identified in the favorable and intermediate risk groups. Although the findings are difficult to interpret, especially with early follow-up for these treatment strategies, future studies may want to explore efficacy endpoints for the three clinical risk subgroups independently.

There are several limitations of the current study that should be acknowledged. First, although NMAs represent a valid and well-suited means for indirect comparisons [42], [43], they cannot replace the gold standard of RCTs. More head-to-head comparisons are required against emerging standard-of-care therapies to confidently identify the optimal treatment strategy. Second, the analysis is limited by the data reported in each of the trials. Some of the outcomes assessed have missing elements that can make interpretation difficult, such as variable reporting of outcomes by IMDC/MSKCC clinical risk subgroups and PRO data. Similarly, we were unable to perform meaningful subgroup analysis because of inconsistent reporting across trials, such as outcomes according to PD-L1 status, location of metastases (bone, visceral), sarcomatoid differentiation, and patients with prior nephrectomy. Third, the data can be affected by clinical heterogeneity between trials such as sample size, PD-L1 status, and relative the proportions of clinical risk groups. Furthermore, there was variable inclusion of IMDC and/or MSKCC clinical risk groups in each of the trials, introducing further clinical heterogeneity. For the purposes of this NMA, a general assumption was made that the IMDC and MSKCC risk group classifications were analogous to permit inclusion of all relevant trials; however, some data suggest that the MSKCC risk classification may be more suitable than the IMDC system in guiding the delivery of immunotherapy checkpoint inhibitors [44], [45]. Furthermore, variations in trial design and the power for primary endpoints can confound the analysis and overall interpretation. In fact, only CLEAR (PEMBRO-LENV) and CheckMate 9ER (NIVO-CABO) were consistently powered for the primary endpoint of PFS, whereas CheckMate 214 (NIVO-IPI) investigated OS/PFS in the intermediate/poor risk group, Keynote 426 evaluated OS in the ITT population, IMmotion 151 (ATEZO-BEV) assessed OS in the group with PD-L1 ≥1%, and Javelin 101 investigated OS in the ITT population and PFS in the group with PD-L1 ≥1%. Subsequent anticancer therapies represent another variable not accounted for that can affect the overall results and analysis. In addition, results were obtained from trials with differing median follow-up, which can make interpretation of the analysis difficult. Finally, as this NMA was focused on first-line IO-TKI combinations, this limited our inclusion of approved TKIs other than sunitinib, such as pazopanib and cabozantinib [8], [46].

## 4 Conclusions

In summary, this systematic review and NMA of first-line treatments for mRCC consistently favored IO-TKI drug combinations over sunitinib monotherapy and dual IO-IO with NIVO-IPI for the primary endpoints of OS and PFS in the ITT population and across all IMDC/MSKCC risk groups. Interestingly, despite a persistent trend favoring IO-TKI for ORR, NIVO-IPI and PEMBRO-LENV demonstrated comparable higher CR rates. IO-TKIs demonstrated the highest rate of grade 3–4 TRAEs but the lowest rate of TRDD, whereas the opposite was true for NIVO-IPI, suggesting a toxicity profile with IO-IO combinations that is difficult to manage. PEMBRO-LENV, NIVO-CABO, and NIVO-IPI were associated with ideal HRQoL outcomes and the lowest risk of treatment-related deterioration. Collectively, there appears to be favorable efficacy and safety with the more recent IO-TKI combinations of PEMBRO-LENV and NIVO-CABO, regardless of clinical risk group. Overall, this NMA supports an emerging role for IO-TKI combinations; however, the durability of this treatment strategy will be further clarified as data mature with longer follow-up. A “living NMA” is in development with the aim of continuously updating new and prior data as they become available. With the rapidly changing treatment landscape for mRCC, a primary goal is to build an online interactive framework to easily navigate the most up-to-date results to guide clinical management.

  ***Author contributions***: Nicholas A. Bosma had full access to all the data in the study and takes responsibility for the integrity of the data and the accuracy of the data analysis.

*Study concept and design*: Bosma, Lee-Ying, Karim.

*Acquisition of data*: Bosma, Gan.

*Analysis and interpretation of data*: Bosma, Warkentin, Brenner.

*Drafting of the manuscript*: Bosma.

*Critical revision of the manuscript for important intellectual content*: Lee-Ying, Karim, Heng, Gan.

*Statistical analysis*: Warkentin, Brenner.

*Obtaining funding*: None.

*Administrative, technical, or material support*: None.

*Supervision*: Lee-Ying, Karim, Heng.

*Other*: None.

  ***Financial disclosures:*** Nicholas A. Bosma certifies that all conflicts of interest, including specific financial interests and relationships and affiliations relevant to the subject matter or materials discussed in the manuscript (eg, employment/affiliation, grants or funding, consultancies, honoraria, stock ownership or options, expert testimony, royalties, or patents filed, received, or pending), are the following: None.

  ***Funding/Support and role of the sponsor*:** None.

  ***Acknowledgments*:** The authors thank Health Sciences and Knowledge Resource librarians Ms. Heather Ganshorn and Mr. Marcus Vaska for their contributions and piloting of our search strategy.

  ***Data sharing statement*:** Any supplementary data, including data extraction forms and templates, supporting the findings of this study are available on request from the corresponding author.
