# Supplementary material for: Efficacy and Safety of First-line Systemic Therapy for Metastatic Renal Cell Carcinoma: A Systematic Review and Network Meta-analysis
Source: Eur Urol Open Sci. 2022 Jan 22;37:14–26. doi: 10.1016/j.euros.2021.12.007 (PMC8792068; doi:10.1016/j.euros.2021.12.007)
Supplement: Supplementary data 1 [file mmc1.docx]

**SUPPLEMENTARY MATERIALS**

**Supplementary Table 1:** PRISMA 2020 Checklist

| **Section and Topic** | **Item #** | **Checklist item** | **Location where item is reported** |
| --- | --- | --- | --- |
| **TITLE** | | |  |
| Title | 1 | Identify the report as a systematic review. | 1 |
| **ABSTRACT** | | |  |
| Abstract | 2 | See the PRISMA 2020 for Abstracts checklist. | 2 |
| **INTRODUCTION** | | |  |
| Rationale | 3 | Describe the rationale for the review in the context of existing knowledge. | 3 |
| Objectives | 4 | Provide an explicit statement of the objective(s) or question(s) the review addresses. | 3 |
| **METHODS** | | |  |
| Eligibility criteria | 5 | Specify the inclusion and exclusion criteria for the review and how studies were grouped for the syntheses. | 4 |
| Information sources | 6 | Specify all databases, registers, websites, organisations, reference lists and other sources searched or consulted to identify studies. Specify the date when each source was last searched or consulted. | 3,4 |
| Search strategy | 7 | Present the full search strategies for all databases, registers and websites, including any filters and limits used. | 3,4,S3-S5 |
| Selection process | 8 | Specify the methods used to decide whether a study met the inclusion criteria of the review, including how many reviewers screened each record and each report retrieved, whether they worked independently, and if applicable, details of automation tools used in the process. | 4 |
| Data collection process | 9 | Specify the methods used to collect data from reports, including how many reviewers collected data from each report, whether they worked independently, any processes for obtaining or confirming data from study investigators, and if applicable, details of automation tools used in the process. | 6,7 |
| Data items | 10a | List and define all outcomes for which data were sought. Specify whether all results that were compatible with each outcome domain in each study were sought (e.g. for all measures, time points, analyses), and if not, the methods used to decide which results to collect. | 4,S6 |
|  | 10b | List and define all other variables for which data were sought (e.g. participant and intervention characteristics, funding sources). Describe any assumptions made about any missing or unclear information. | 4,S6 |
| Study risk of bias assessment | 11 | Specify the methods used to assess risk of bias in the included studies, including details of the tool(s) used, how many reviewers assessed each study and whether they worked independently, and if applicable, details of automation tools used in the process. | 4,5 |
| Effect measures | 12 | Specify for each outcome the effect measure(s) (e.g. risk ratio, mean difference) used in the synthesis or presentation of results. | 5 |
| Synthesis methods | 13a | Describe the processes used to decide which studies were eligible for each synthesis (e.g. tabulating the study intervention characteristics and comparing against the planned groups for each synthesis (item #5)). | 5,S6 |
|  | 13b | Describe any methods required to prepare the data for presentation or synthesis, such as handling of missing summary statistics, or data conversions. | 5 |
|  | 13c | Describe any methods used to tabulate or visually display results of individual studies and syntheses. | 5 |
|  | 13d | Describe any methods used to synthesize results and provide a rationale for the choice(s). If meta-analysis was performed, describe the model(s), method(s) to identify the presence and extent of statistical heterogeneity, and software package(s) used. | 5 |
|  | 13e | Describe any methods used to explore possible causes of heterogeneity among study results (e.g. subgroup analysis, meta-regression). | 5 |
|  | 13f | Describe any sensitivity analyses conducted to assess robustness of the synthesized results. | 5 |
| Reporting bias assessment | 14 | Describe any methods used to assess risk of bias due to missing results in a synthesis (arising from reporting biases). | 4,5 |
| Certainty assessment | 15 | Describe any methods used to assess certainty (or confidence) in the body of evidence for an outcome. | 4,5 |
| **RESULTS** | | |  |
| Study selection | 16a | Describe the results of the search and selection process, from the number of records identified in the search to the number of studies included in the review, ideally using a flow diagram. | 4,5,16 |
|  | 16b | Cite studies that might appear to meet the inclusion criteria, but which were excluded, and explain why they were excluded. | 16 |
| Study characteristics | 17 | Cite each included study and present its characteristics. | 17 |
| Risk of bias in studies | 18 | Present assessments of risk of bias for each included study. | S7 |
| Results of individual studies | 19 | For all outcomes, present, for each study: (a) summary statistics for each group (where appropriate) and (b) an effect estimate and its precision (e.g. confidence/credible interval), ideally using structured tables or plots. | 18-21 |
| Results of syntheses | 20a | For each synthesis, briefly summarise the characteristics and risk of bias among contributing studies. | 5,6 |
|  | 20b | Present results of all statistical syntheses conducted. If meta-analysis was done, present for each the summary estimate and its precision (e.g. confidence/credible interval) and measures of statistical heterogeneity. If comparing groups, describe the direction of the effect. | 18-21 |
|  | 20c | Present results of all investigations of possible causes of heterogeneity among study results. | 8 |
|  | 20d | Present results of all sensitivity analyses conducted to assess the robustness of the synthesized results. | 6-8,18-21 |
| Reporting biases | 21 | Present assessments of risk of bias due to missing results (arising from reporting biases) for each synthesis assessed. | 6-8 |
| Certainty of evidence | 22 | Present assessments of certainty (or confidence) in the body of evidence for each outcome assessed. | 6-8 |
| **DISCUSSION** | | |  |
| Discussion | 23a | Provide a general interpretation of the results in the context of other evidence. | 8-12 |
|  | 23b | Discuss any limitations of the evidence included in the review. | 11,12 |
|  | 23c | Discuss any limitations of the review processes used. | 11 |
|  | 23d | Discuss implications of the results for practice, policy, and future research. | 11,12 |
| **OTHER INFORMATION** | | |  |
| Registration and protocol | 24a | Provide registration information for the review, including register name and registration number, or state that the review was not registered. | Not registered |
|  | 24b | Indicate where the review protocol can be accessed, or state that a protocol was not prepared. | No formal protocol |
|  | 24c | Describe and explain any amendments to information provided at registration or in the protocol. | N/A |
| Support | 25 | Describe sources of financial or non-financial support for the review, and the role of the funders or sponsors in the review. | 1 |
| Competing interests | 26 | Declare any competing interests of review authors. | 1 |
| Availability of data, code and other materials | 27 | Report which of the following are publicly available and where they can be found: template data collection forms; data extracted from included studies; data used for all analyses; analytic code; any other materials used in the review. | 1 |

**Supplementary Table 2.** Medline Search Strategy

Database: Ovid MEDLINE(R) and Epub Ahead of Print, In-Process & Other Non-Indexed Citations and Daily <1946 to March 25, 2021>

Search Strategy:

--------------------------------------------------------------------------------

1 first line therap*.ab,ti. (16903)

2 "systemic therap* ".ab,ti. (17152)

3 "first line systemic therap* ".ab,ti. (237)

4 "first line treat* ".ab,ti. (25956)

5 (first* adj3 "line systemic therap*").mp. (254)

6 (first* adj3 line treat*).mp. (26670)

7 "tyrosine kinase inhibitor* ".ab,ti. (29469)

8 TKI.ab,ti. (8289)

9 exp Protein Kinase Inhibitors/ (102764)

10 exp Protein-Tyrosine Kinases/ (222362)

11 "small-molecule inhibitor* ".ab,ti. (13507)

12 "monoclonal antibod* ".ab,ti. (191513)

13 exp Antibodies, Monoclonal/ (248208)

14 "immune checkpoint inhibitor* ".ab,ti. (10533)

15 "immune checkpoint blockade* ".ab,ti. (3100)

16 (immune* adj3 inhibitor*).mp. (14675)

17 (immune* adj3 blockade*).mp. (3871)

18 exp immunotherapy/ (289741)

19 exp Sunitinib/ (3723)

20 sunitinib.ab,ti. (5896)

21 sutent.ab,ti. (153)

22 pazopanib.ab,ti. (1780)

23 cabozantinib.ab,ti. (972)

24 axitinib.ab,ti. (1004)

25 exp Axitinib/ (568)

26 lenvatinib.ab,ti. (967)

27 tivozanib.ab,ti. (116)

28 sorafenib.ab,ti. (8883)

29 exp Sorafenib/ (5211)

30 temsirolimus.ab,ti. (1277)

31 exp temsirolimus/ (0)

32 exp Everolimus/ (5006)

33 everolimus.ab,ti. (6808)

34 bevacizumab.ab,ti. (16913)

35 exp Bevacizumab/ (12286)

36 exp Nivolumab/ (3241)

37 nivolumab.ab,ti. (5508)

38 ipilumumab.ab,ti. (20)

39 exp ipilimumab/ (2172)

40 pembrolizumab.ab,ti. (4515)

41 exp pembrolizumab/ (0)

42 avelumab.ab,ti. (501)

43 exp avelumab/ (0)

44 atezolizumab.ab,ti. (1300)

45 exp atezolizumab/ (0)

46 "interferon*".ab,ti. (158807)

47 exp interferon/ (137885)

48 IFN.ab,ti. (123571)

49 "interleukin*".ab,ti. (249683)

50 IL.ab,ti. (365087)

51 "antineoplastic agent* ".ab,ti. (5439)

52 exp Antineoplastic Agents/ (1144038)

53 "antineoplastic target* ".ab,ti. (38)

54 "antineoplastic drug* ".ab,ti. (3354)

55 "chemotherap*".ab,ti. (430592)

56 "drug therap* ".ab,ti. (40991)

57 exp chemotherapy/ (1407550)

58 exp Drug Therapy/ (1407550)

59 exp Molecular Targeted Therapy/ (31358)

60 "IFN alpha".ab,ti. (17143)

61 "interferon alpha".ab,ti. (19576)

62 exp Interferon-alpha/ (29163)

63 "anticancer agent* ".ab,ti. (20133)

64 "anticancer drug* ".ab,ti. (30376)

65 "anticancer target* ".ab,ti. (808)

66 "anticarcino* agent* ".ab,ti. (157)

67 "anticarcino* drug* ".ab,ti. (36)

68 "anticarcino* target* ".ab,ti. (1)

69 "antitumour* target* ".ab,ti. (3)

70 "antitumour* drug* ".ab,ti. (487)

71 "antitumour* agent* ".ab,ti. (772)

72 "antitumor* drug* ".ab,ti. (5301)

73 "antitumor* target* ".ab,ti. (152)

74 "immunotherap*".ab,ti. (95683)

75 1 or 2 or 3 or 4 or 5 or 6 or 7 or 8 or 9 or 10 or 11 or 12 or 13 or 14 or 15 or 16 or 17 or 18 or 19 or 20 or 21 or 22 or 23 or 24 or 25 or 26 or 27 or 28 or 29 or 30 or 31 or 32 or 33 or 34 or 35 or 36 or 37 or 38 or 39 or 40 or 41 or 42 or 43 or 44 or 45 or 46 or 47 or 48 or 49 or 50 or 51 or 52 or 53 or 54 or 55 or 56 or 57 or 58 or 59 or 60 or 61 or 62 or 63 or 64 or 65 or 66 or 67 or 68 or 69 or 70 or 71 or 72 or 73 or 74 (3585425)

76 "renal cell carcinoma".ab,ti. (37701)

77 "advanced renal cell carcinoma".ab,ti. (1839)

78 exp Carcinoma, Renal Cell/ (34978)

79 "metastatic renal cell carcinoma".ab,ti. (6101)

80 "kidney cancer".ab,ti. (4797)

81 "kidney neoplasm* ".ab,ti. (275)

82 exp Kidney Neoplasms/ (76995)

83 "kidney carcinoma".ab,ti. (342)

84 "kidney tumor* ".ab,ti. (2350)

85 "kidney tumour* ".ab,ti. (437)

86 "advanced kidney cancer".ab,ti. (127)

87 "metastatic kidney cancer".ab,ti. (192)

88 "renal cancer".ab,ti. (5956)

89 "renal carcinoma".ab,ti. (5037)

90 "renal neoplasm* ".ab,ti. (1404)

91 "renal tumor* ".ab,ti. (8064)

92 "renal tumour* ".ab,ti. (1807)

93 (kidney adj3 adenocarcinoma).mp. (362)

94 (renal adj3 adenocarcinoma).mp. (1487)

95 (hypernephroid adj3 carcinoma*).mp. (116)

96 (nephroid adj3 carcinoma*).mp. (0)

97 ("collecting duct" adj3 carcinoma*).mp. (402)

98 (renal adj3 carcinoma*).mp. (53319)

99 (kidney adj3 carcinoma*).mp. (2155)

100 76 or 77 or 78 or 79 or 80 or 81 or 82 or 83 or 84 or 85 or 86 or 87 or 88 or 89 or 90 or 91 or 92 or 93 or 94 or 95 or 96 or 97 or 98 or 99 (95607)

101 "survival analysis".ab,ti. (32197)

102 exp Survival Analysis/ (312804)

103 exp Survival/ (4818)

104 survival.ab,ti. (1009757)

105 "disease-free survival".ab,ti. (44716)

106 exp Disease-Free Survival/ (77484)

107 "progression-free survival* ".ab,ti. (50810)

108 "survival rate* ".ab,ti. (157338)

109 exp Survival Rate/ (181355)

110 (rate* adj3 survival).mp. (318524)

111 exp Survivors/ (34524)

112 "survivor*".ab,ti. (109592)

113 "overall survival".ab,ti. (185418)

114 "cancer survivor* ".ab,ti. (18971)

115 exp Cancer Survivors/ (5228)

116 "outcome*".ab,ti. (1898646)

117 "treatment outcome* ".ab,ti. (53459)

118 exp Treatment Outcome/ (1117112)

119 exp Disease Progression/ (191230)

120 "disease progression".ab,ti. (82768)

121 "adverse event* ".ab,ti. (176701)

122 "adverse outcome* ".ab,ti. (32101)

123 exp Adverse Outcome Pathways/ (159)

124 exp Long Term Adverse Effects/ (674)

125 exp "Drug-Related Side Effects and Adverse Reactions"/ (120654)

126 (rate* adj3 response).mp. (137846)

127 exp Safety/ (84120)

128 "response rate* ".ab,ti. (121831)

129 safety.ab,ti. (554893)

130 101 or 102 or 103 or 104 or 105 or 106 or 107 or 108 or 109 or 110 or 111 or 112 or 113 or 114 or 115 or 116 or 117 or 118 or 119 or 120 or 121 or 122 or 123 or 124 or 125 or 126 or 127 or 128 or 129 (4207142)

131 75 and 100 and 130 (11374)

132 ((randomized controlled trial or controlled clinical trial).pt. or (Randomized or placebo or randomly or trial or groups).ab. or drug therapy.fs.) not (exp animals/ not exp humans/) (4373030)

133 (randomi#ed adj3 study).mp. (107855)

134 (randomi#ed adj3 trial).mp. (619526)

135 132 or 133 or 134 (4398924)

136 131 and 135 (6873)

137 limit 136 to (english language and yr="2000 -Current") (5676)

138 limit 137 to (clinical trial, phase iii or randomized controlled trial) (413)

***************************

**Supplementary Table 3.** Summary of data extraction variables from included studies

| Study Information | Author list  Title  Year of publication (original and updates)  Journal  Trial name  Inclusion/Exclusion criteria of trial  Randomization design  Primary and Secondary endpoints of trial  MSKCC or IMDC risk criteria used  Randomization period |
| --- | --- |
| Eligibility Checklist | Original data (Y/N)  First-line treatment for advanced or mRCC (Y/N)  First-line treatment included IO-drug combinations (Y/N)  Data for ITT and/or MSKCC/IMDC risk groups (Y/N)  Reports on efficacy outcomes (OS/PFS/ORR/CR) (Y/N) and/or  Reports on safety outcomes (Y/N) and/or  Reports on health-related quality of life (Y/N)  Human Subjects (Y/N)  Randomized Controlled Trial (Y/N)  Years 2000-Current |
| Study Details | Sample size (ITT and risk groups)  Intervention and control arm  Median follow-up |
| Patient Demographics | Median age  Female (%)/Male (%)  Sites of metastatic disease (%)  Previous nephrectomy (%)  PDL1 ≥1% and <1% (%)  Subsequent Anticancer Treatment (%) |
| Treatment Characteristics | Drug Regimen (doses)  Treatment Duration |
| Outcome Measures | OS (HR, 95% CI)  PFS (HR, 95% CI)  ORR (%,95% CI)  CR (%, 95% CI)  Median Duration of Response (months, 95% CI)  Any Treatment-related Adverse Events  Treatment-related grade ¾ Adverse Events  Treatment-related drug discontinuation  Health-related quality of life/Patient Reported Outcomes (FKSI, EQ 5D) |

**Supplementary Table 4.** Risk of bias assessment using the Cochrane Collaboration Tool for Assessment of Bias in Randomized Trials

| First Author  Year  Trial Name | Motzer  2018  Checkmate 214 | Motzer  2019  Javelin 101 | Choeiri  2021  Checkmate 9ER | Rini  2019  Keynote 426 | Rini  2019  IMmotion 151 | Motzer  2021  CLEAR |
| --- | --- | --- | --- | --- | --- | --- |
| Random Sequence Generation | 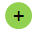 | 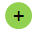 | 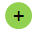 | 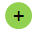 | 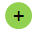 | 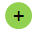 |
| Allocation Concealment | 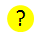 | 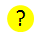 | 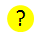 | 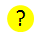 | 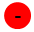 | 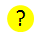 |
| Blinding of Participants | 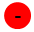 | 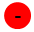 | 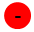 | 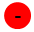 | 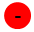 | 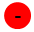 |
| Blinding of Outcome Assessment | 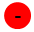 | 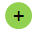 | 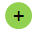 | 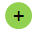 | 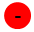 | 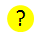 |
| Incomplete Outcome Data | 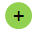 | 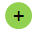 | 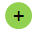 | 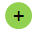 | 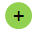 | 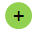 |
| Selective Outcome Reporting | 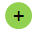 | 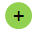 | 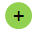 | 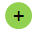 | 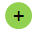 | 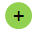 |


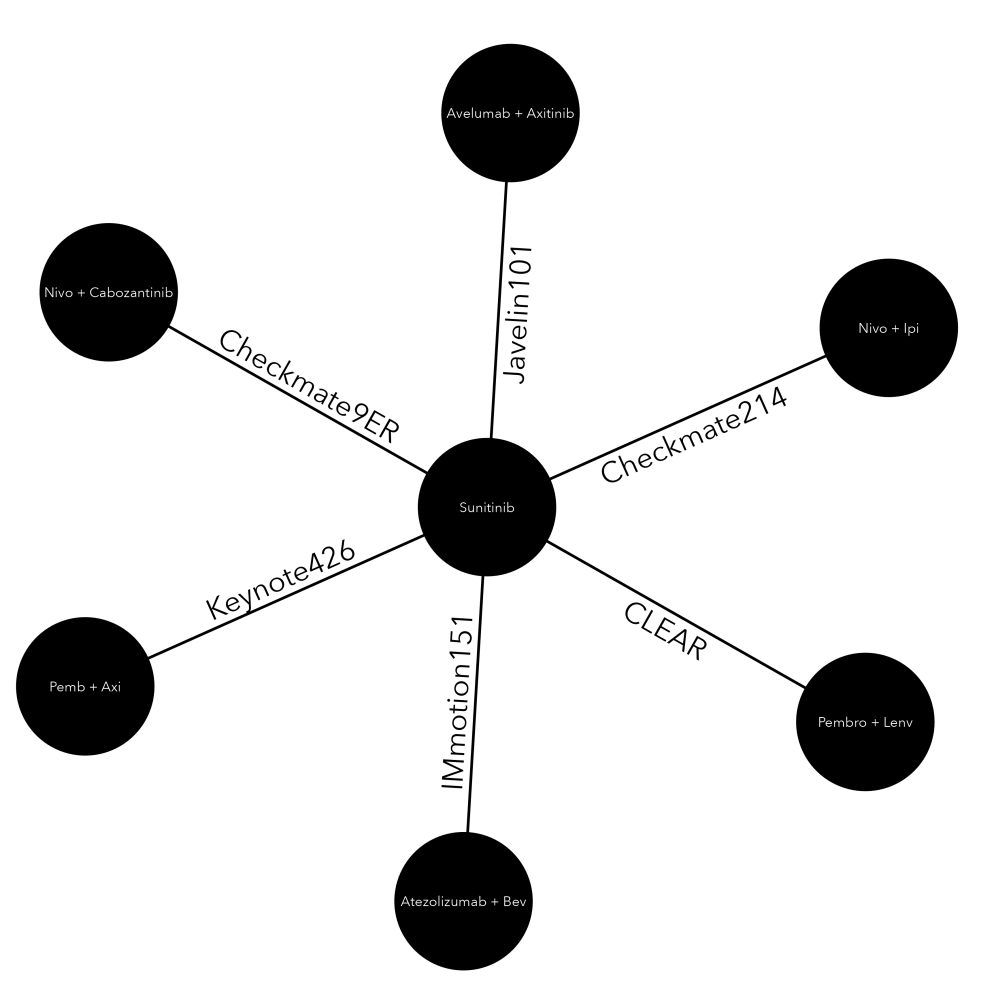


**Supplementary Figure 1.** Network diagram of eligible treatment comparisons. All trials had a common comparator to sunitinib, represented graphically as the center of the network plot.


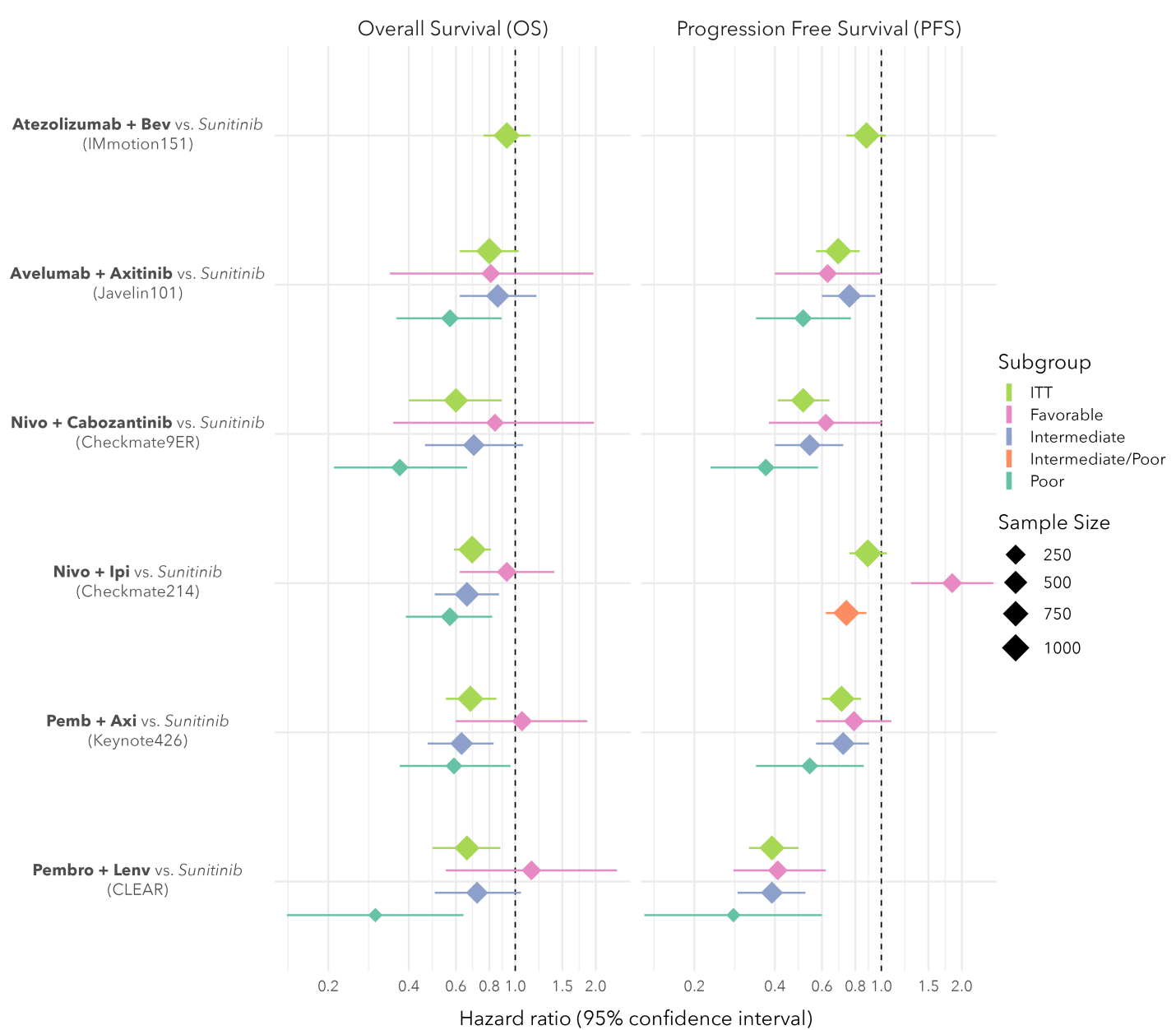


**Supplementary Figure 2.** Summary of HR results of the included trials for OS and PFS by ITT and clinical risk group.


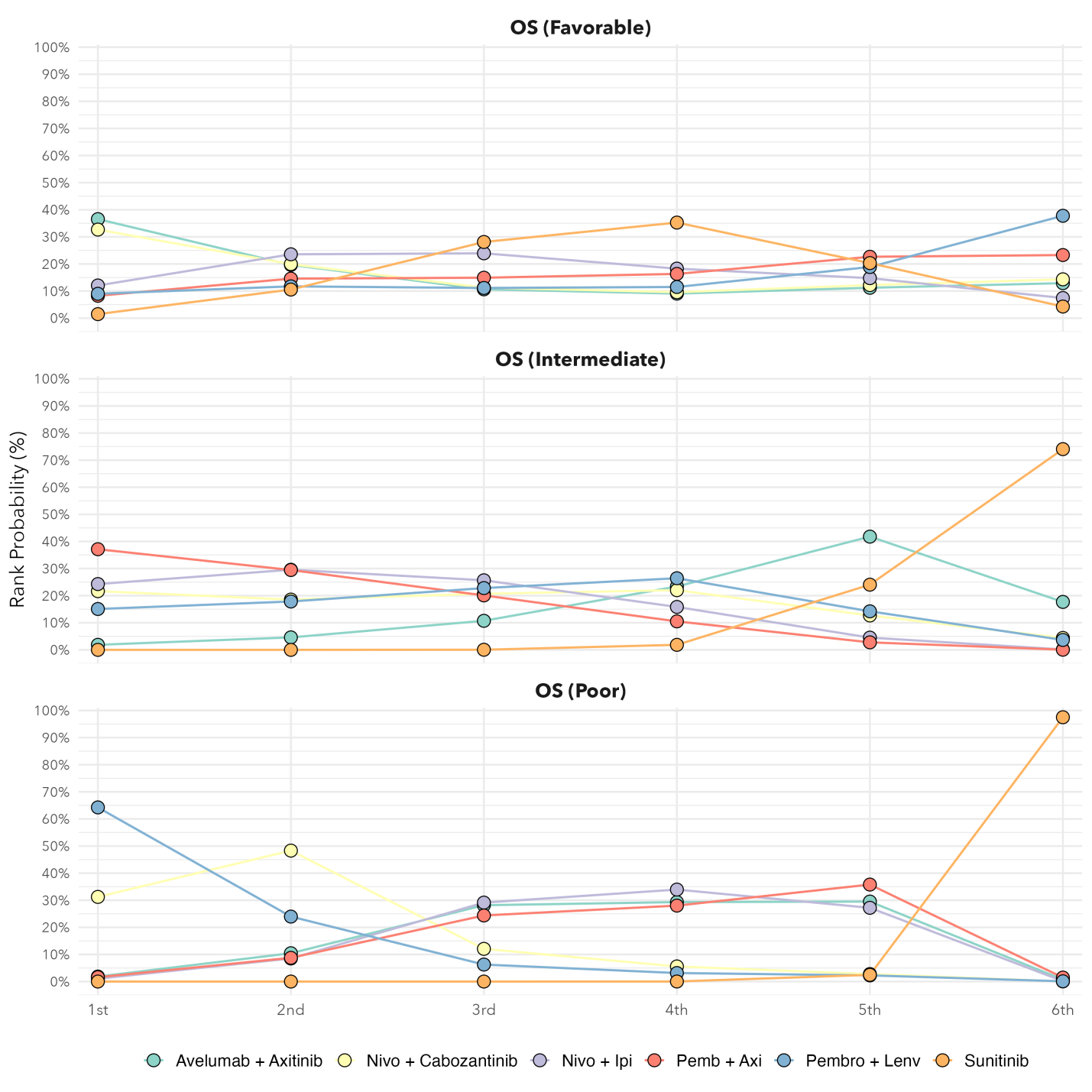


**Supplementary Figure 3**. Rankograms of OS by favorable, intermediate and poor risk group.


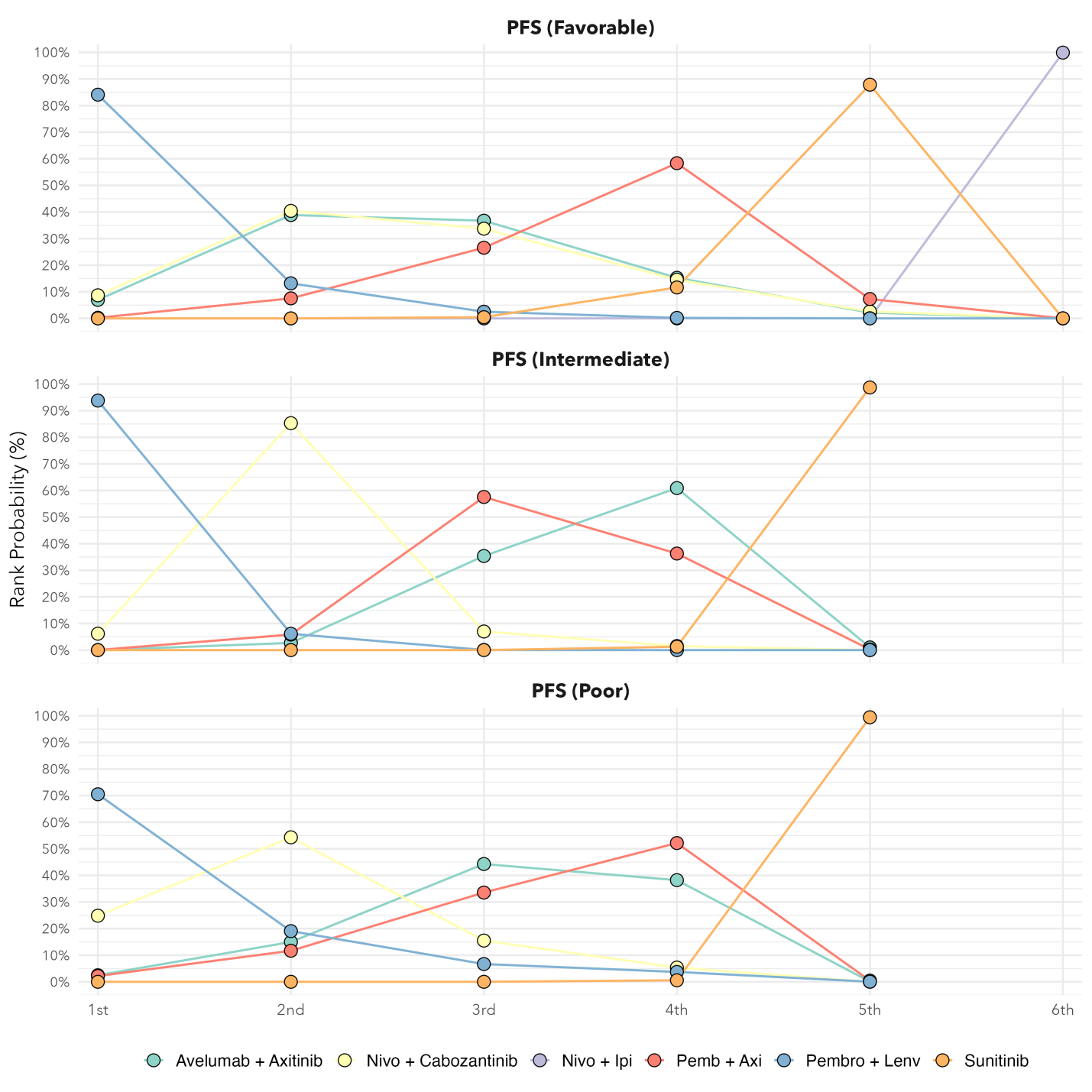


**Supplementary Figure 4.** Rankograms of PFS by favorable, intermediate and poor risk group.


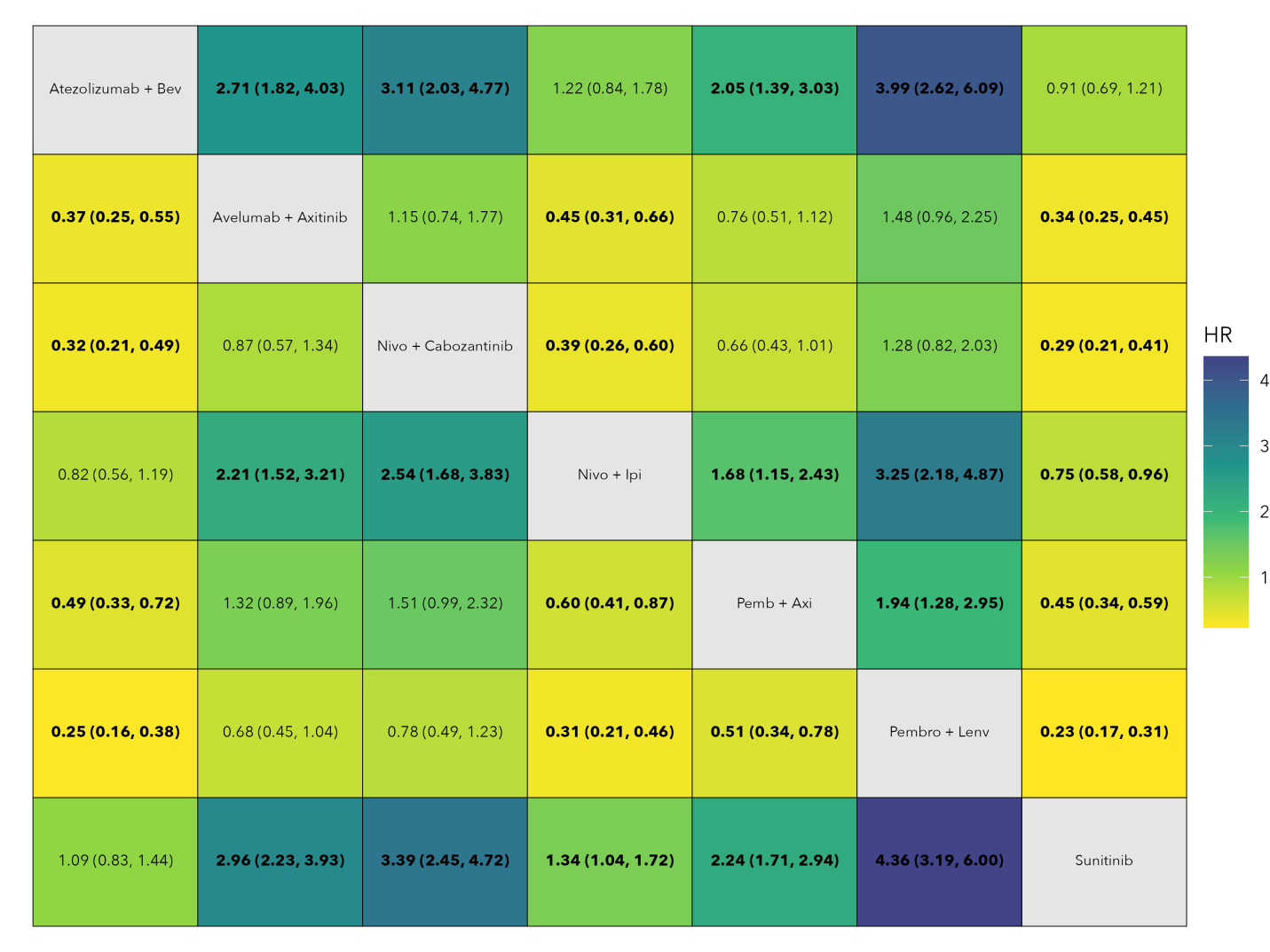


**Supplementary Figure 5**. Overall response rate (ORR). Hazard ratios (HRs) for ORR in the ITT population are represented to allow comparisons of all treatments. Bolded values are statistically significant.


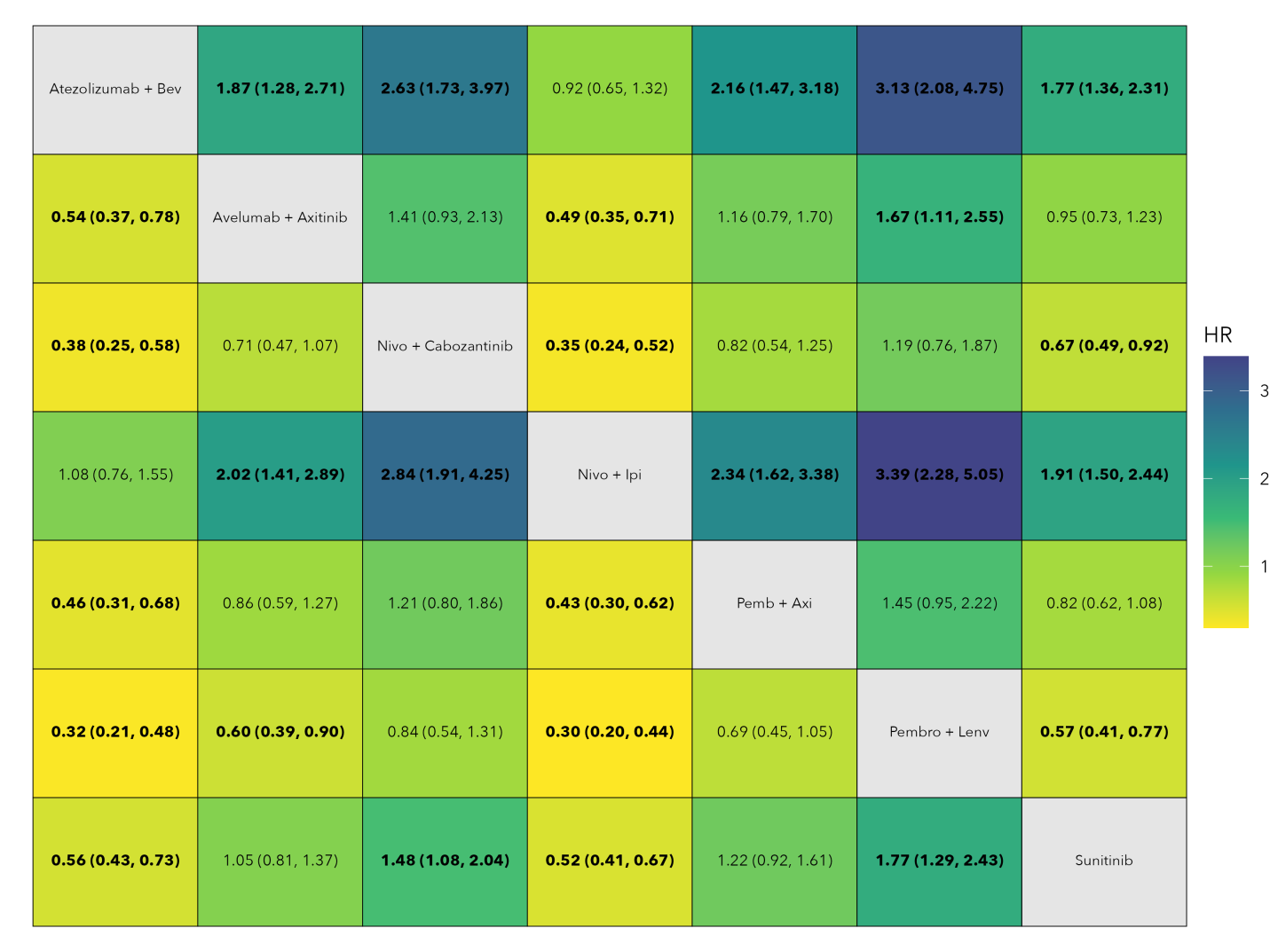


**Supplementary Figure 6**. Treatment-related grade ¾ adverse events in the ITT population. Hazard ratios (HRs) are represented to allow comparisons of all treatments. Bolded values are statistically significant.


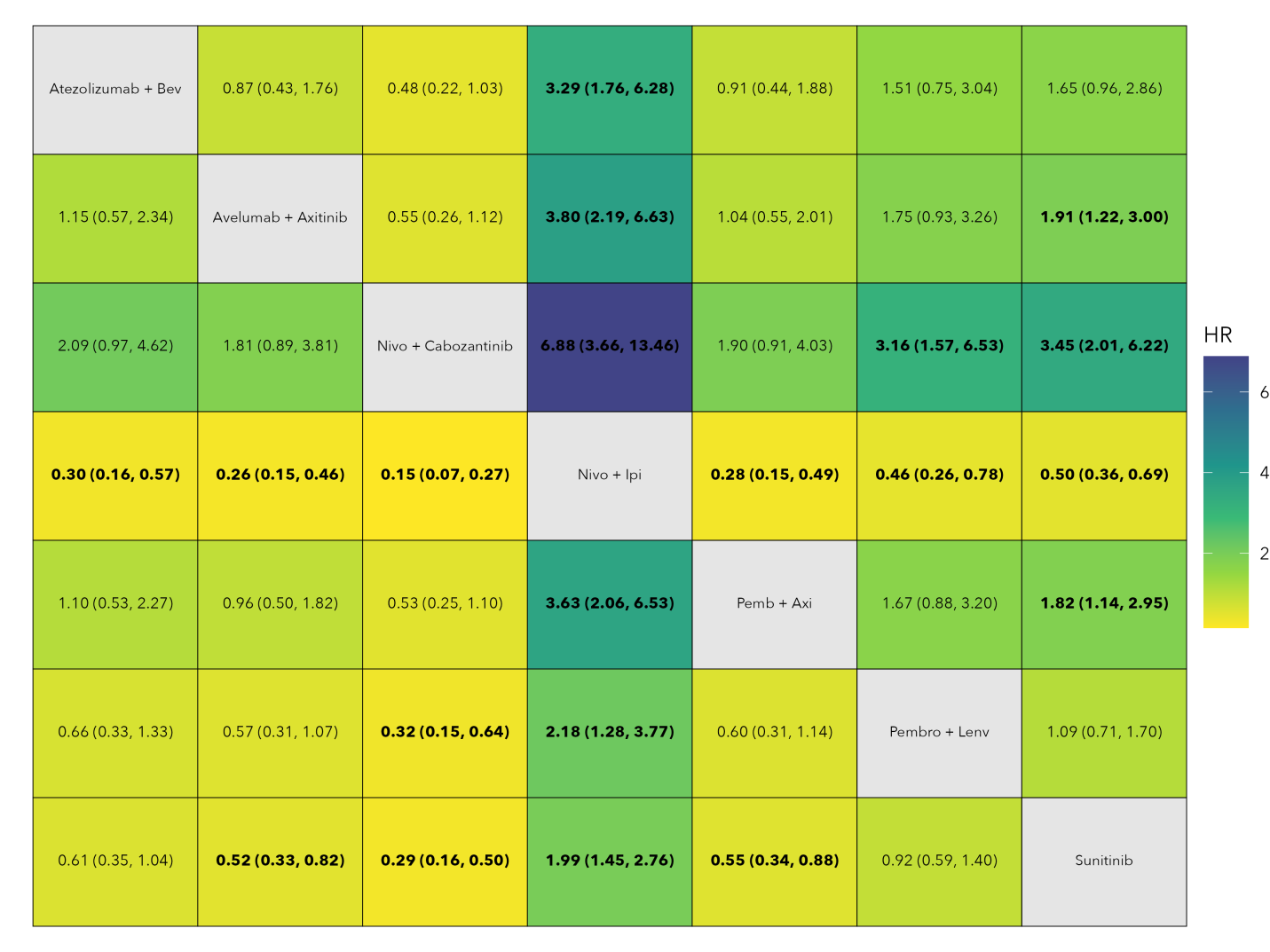


**Supplementary Figure 7**. Treatment-related drug discontinuation in the ITT population. Hazard ratios (HRs) are represented to allow comparisons of all treatments. Bolded values are statistically significant.


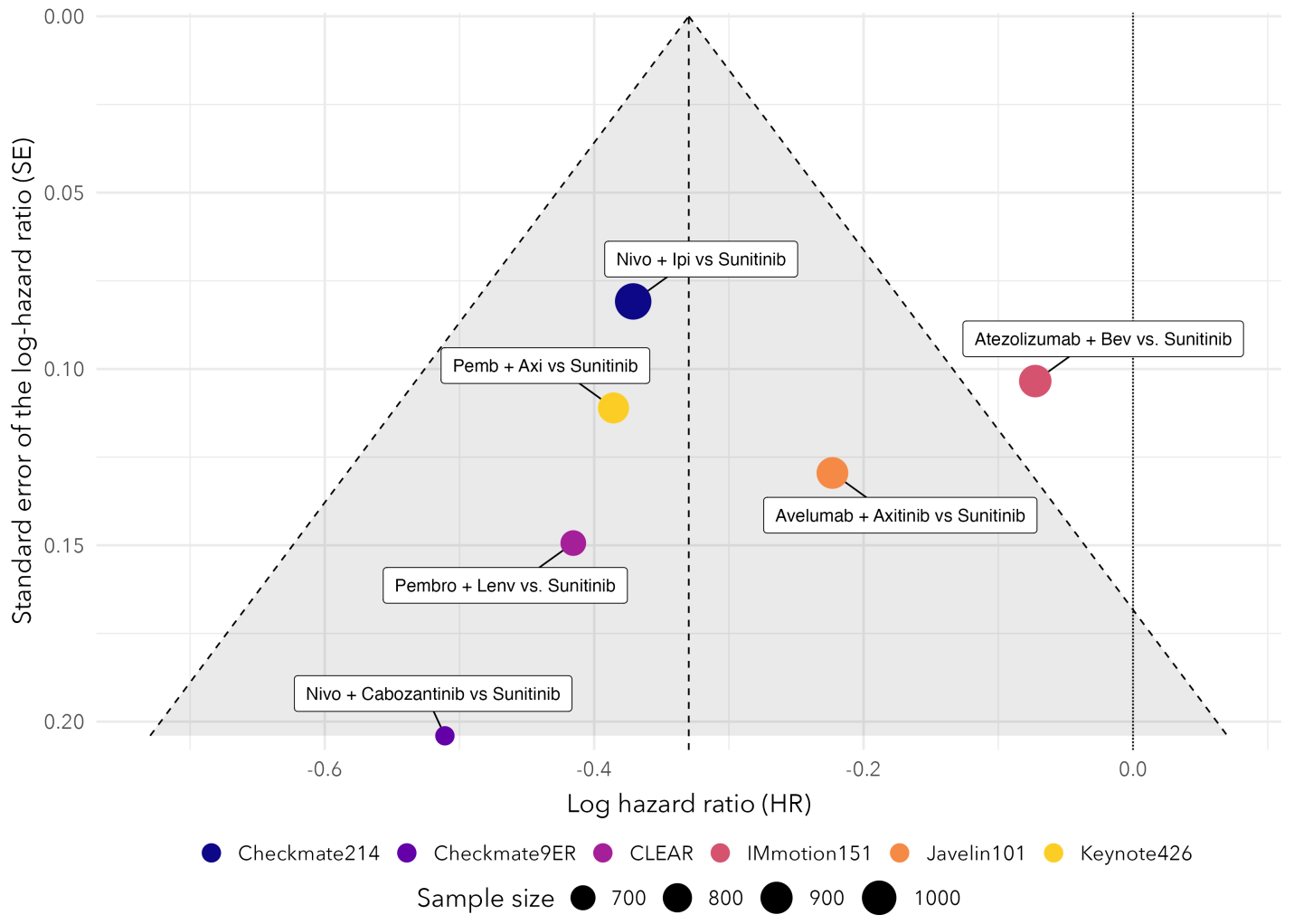


**Supplementary Figure 8.** Funnel plot of included trials for assessment of publication bias.


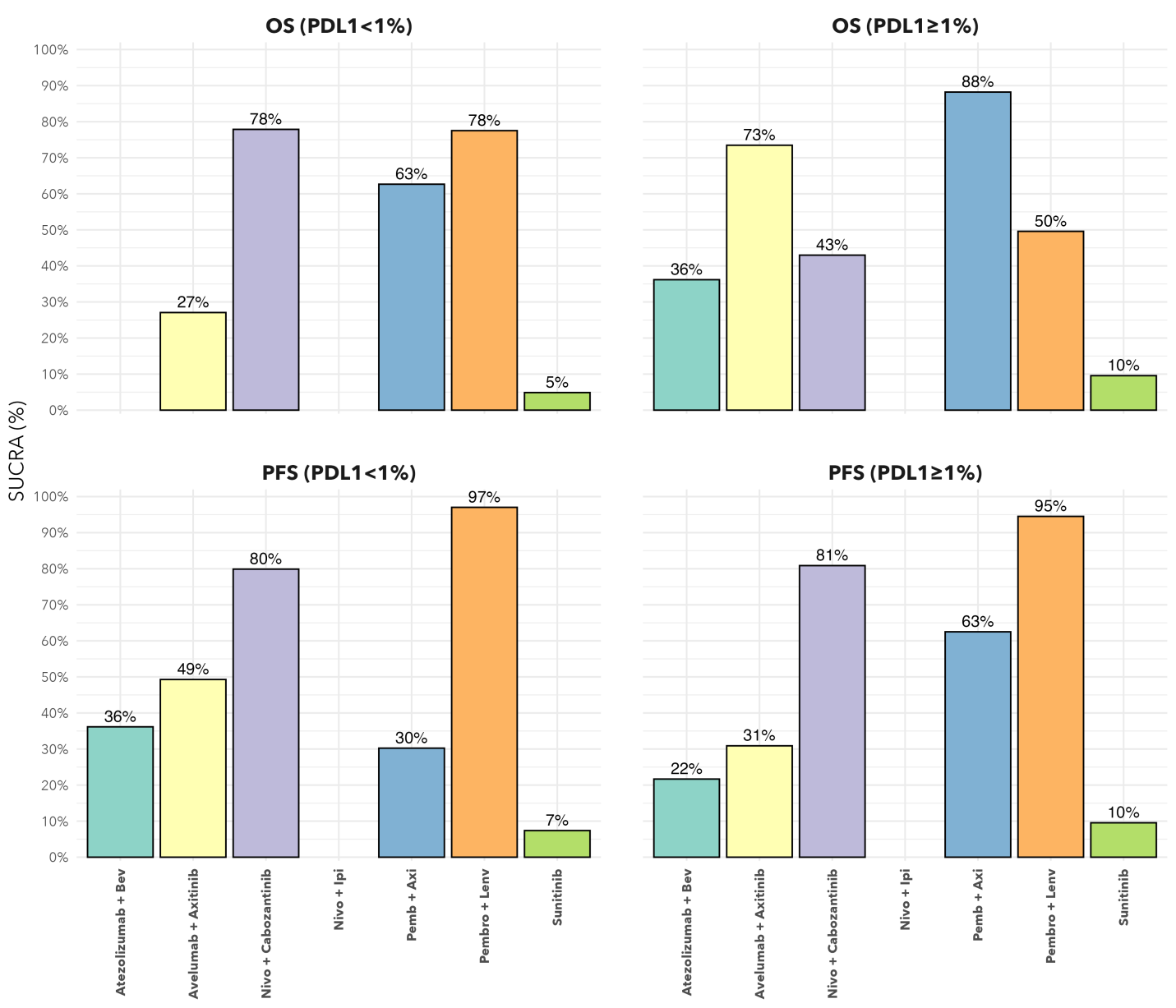


**Supplementary Figure 9.** OS and PFS by PDL1 ≥1% and PDL1 <1% in the overall population. Data for NIVO-IPI was not available in the overall population, as the analysis in the trial was only completed in the intermediate/poor risk group.
